# Future projections of temperature-related indices in Prince Edward Island using ensemble average of three CMIP6 models

**DOI:** 10.1038/s41598-024-63450-9

**Published:** 2024-06-03

**Authors:** Junaid Maqsood, Xiuquan Wang, Aitazaz A. Farooque, Rana Ali Nawaz

**Affiliations:** 1 Applied Research Department, Holland College, Charlottetown, PE C1A 4Z1 Canada; 2 Canadian Centre for Climate Change and Adaptation, University of Prince Edward Island, St. Peter’s Bay, PE C0A 2A0 Canada; 3 School of Climate Change and Adaptation, University of Prince Edward Island, Charlottetown, PE C1A 4P3 Canada; 4 Faculty of Sustainable Design Engineering, University of Prince Edward Island, Charlottetown, PE C1A 4P3 Canada

**Keywords:** Climate change, Environmental impact

## Abstract

Prince Edward Island (PEI) is an agricultural province heavily relying on rainfed agriculture. The island has already experienced significant impacts from climate change. Accurate projections of PEI temperature extreme indices are required to mitigate and adapt to the changing climate conditions. This study aims to develop ensemble projections using Coupled Model Intercomparison Project Phase 6 (CMIP6) global circulation models (GCMs) to analyze temperature extremes on PEI. In this study, the ECMWF ERA5 reanalysis dataset was chosen for stepwise cluster analysis (SCA) due to its high accuracy. Three CMIP6 (NorESM2-MM, MPI-ESM1.2-HR, and CanESM5) GCMs, along with their ensemble average, were utilized in the SCA model to project future changes in daily maximum temperature (Tmax) and minimum temperature (Tmin) at four meteorological stations on PEI (East Point, Charlottetown, Summerside, and North Cape) under two shared socioeconomic pathways (SSP2-4.5 and SSP5-8.5). These GCMs were selected based on their low, medium, and high Equilibrium Climate Sensitivity. The bias-corrected results for the future period of Tmax and Tmin showed that the GCM-specific changes in the ECS also impact the regional scale. Additionally, several temperature extreme indices, including the daily temperature range (DTR), summer days (SU), growing degree days (GDD), growing season length (GSL), ice days (ID), and frost days (FD), were analyzed for two future periods: FP1(202–2050) and FP2 (2051–2075). The results indicate that DTR, SU, GDD, and GSL are expected to increase, while ID and FD are projected to decrease during FP1 and FP2 under both scenarios. The future projected mean monthly changes in Tmax, Tmin, and the selected temperature extreme indices highlight warmer future periods and an increase in agriculture-related indices such as GDD and GSL. Specifically, July, August, and September are expected to experience even higher temperatures in the future. As the climate becomes warmer, cold extreme events are projected to be shorter in duration but more intense in terms of their impact. The largest increments/decrements for Tmax, Tmin, and their relevant indices were observed during FP2 under SSP5-8.5. The outcomes of this study provide valuable insights for agricultural development, water resource management, and the formulation of effective mitigation strategies to address the impacts of climate change on PEI.

## Introduction

The future can witness fluctuations in the intensity and frequency of extreme climatic events, as evidenced by the global average surface temperature of 2011–2020, which surpassed 0.2–1 °C compared to the average temperature recorded from 1850 to 1900^1^. The rise in atmospheric greenhouse gases is primarily responsible for the temperature increase and global warming phenomenon^2^. The shift in the pattern of climatic parameters has also been observed in Canada^3^, including Prince Edward Island (PEI), Canada^4^. Global climate variability has significantly affected various aspects of life, such as agriculture, water resources, and food security^5,6^. Consequently, it becomes essential to thoroughly investigate and comprehend future climate variability at a site-specific scale to adapt to and mitigate these changes.

Reliable information on possible changes in future climate is vital for planning sustainable development. In this regard, global circulation models (GCMs) are considered valuable tools for identifying historical and projected climate variations, and their outcomes have been extensively employed for various purposes. The first phase of CMIP started 20 years ago under the guidance of the World Climate Research Program Working Group on Paired Models. Over its six phases (CMIP6), CMIP has played a crucial role in developing and evaluating models that progressively offer comprehensive representations of the climate system^7^. CMIP6 was developed by integrating common socioeconomic trajectories with trajectories that represent greenhouse gas concentrations. This integration allows for examining the feedback between climate change and socioeconomic factors, including global population growth, economic development, and technological advancements^8^. The shared socioeconomic pathway (SSP) covers various economic and social domains^9^. The CMIP6 covers a wide range of updated models and eliminates some of the limitations and uncertainties of CMIP5. The CMIP6 launch has led to extensive studies on the comparative evaluation of CMIP6 and CMIP5 models in simulating temperature and precipitation extremes. Hamed et al.^10^ compare the CMIP6 and CMIP5 models to simulate the precipitation and temperature in Egypt. Their results revealed that CMIP6 models better replicated the historical Tmax, Tmin, and precipitation, and CMIP5 models exhibited a higher bias than CMIP6. Zhou et al.^11^ also compared the CMIP6 and CMIP5 models to project the surface air temperature of the Tibetan Plateau. They found less uncertainty in the CMIP6 results compared to CMIP5. The future projection results of the CMIP5 model (CanESM2) for Tmax and Tmin showed that the increments in these climatic parameters for the growing season in PEI are expected 0.72–5.37 °C and 0.87–5.91 °C, respectively, irrespective of the RCPs^4^. So, there is a need to analyze the performance of CMIP6 models for future periods of PEI.

However, the outputs of GCMs frequently exhibit limitations in terms of temporal and spatial resolution, leading to systematic biases and increased uncertainties in projected climate variables. To address these challenges, utilizing multiple-model ensembles (MME) of GCM models is common practice, allowing for a more comprehensive assessment of the projected climate conditions^12,13^. Comparison studies also indicated that CMIP6 models performed better regarding MME than CMIP5 models^14,15^. GCMs are globally used to simulate climatic variables but cannot be directly used due to coarse spatial resolution^16^. Therefore, downscaling techniques are necessary to enhance the resolution of these model outputs and align them with local scales. Statistical and dynamic downscaling methods have been developed to achieve this transformation^17,18^. In dynamic downscaling, GCMs simulate regional climate models locally by incorporating detailed physical processes and boundary conditions^19^. This technique can provide higher spatial resolution based on coarse-scale GCM outputs, but it requires substantial data and computational resources, which may exceed the scope of many studies. On the other hand, a statistical relationship is established between observed climate variables and GCM outputs to develop predictand-predictor relationships^20^. The statistical downscaling also has high accuracy in downscaling the temperature data^4^. This approach offers advantages in terms of computational efficiency and accuracy compared to dynamic downscaling. During statistical downscaling, calibration or training periods aim to replicate historical regional climatic parameters. This Statistical downscaling technique is widely adopted due to its simplicity and lower computational requirements than dynamic downscaling methods.

In statistical downscaling, climate science researchers have widely used reanalysis data to downscale coarse-resolution GCM data to regional scales^21^. These datasets are prepared using the spectral statistical interpolation method, which incorporates various sources such as national archives, meteorological observation stations, ship and aircraft observations, satellite data, and weather forecasting models^22^. Selecting the appropriate reanalysis dataset is crucial for obtaining accurate outputs of current and future rainfall and temperature variables in regions affected by climate change. Nacar et al.^23^ compared the performance of downscaling models with three different reanalysis datasets (NCEP/NCAR, ERA-Interim, and ERA5), and it was revealed that the simulated values by ERA5 models were in closer agreement with the observed climatic. This conclusion was drawn based on the lower RMSE values obtained from the ERA5 models compared to those from NCEP and ERA-Interim. The RMSE values observed across all 36 selected stations in this study varied between 0.35 and 0.79. Furthermore, recent downscaling studies have also utilized the ERA5 and NCEP-DOE reanalysis 2 datasets^24,25^. However, the studies comparing these updated reanalysis datasets are limited.

Numerous statistical downscaling methods exist, but the relationship between large-scale and small-scale climatic parameters can be discrete and highly non-linear. Conventional statistical downscaling methods assume explicit functional expressions, which may not be suitable in such cases. Consequently, there is a need for more effective approaches to address this complexity. Stepwise cluster analysis (SCA) is one of those approaches that can handle discrete or continuous, linear, or non-linear variables without relying on a predefined functional relationship. SCA is a multivariate automatic interaction detection algorithm initially developed by Huang^26^ for air quality modeling. Due to the superior performance of SCA, it has applications in various domains, including groundwater modeling, air quality management, and climate modeling^27–29^. Therefore, in this study, SCA is utilized for the statistical downscaling of temperature at various PEI stations. Zhai et al.^30^ also observed the high accuracy of the SCA model with MME outputs to downscale and future projects the Tmax, Tmin, and Tmean in Ottawa, Canada. After downscaling, the uncertainties present in the model’s simulated results can be removed using different bias correction methods. Different comparison studies showed that the quantile delta mapping method performs well in removing the biases from the model simulated results compared to the other methods, such as the delta method, quantile mapping, linear scaling, and scaled distribution mapping^31,32^.

Therefore, this research aims to develop a stepwise clustered downscaling model to help investigate the plausible changes in Tmax and Tmin in PEI. The main aim of this paper is to (i) evaluate reanalysis datasets (NCEP-DOE Reanalysis 2 and ECMWF ERA5) that can complement the observed dataset, (ii) three GCMs (i.e., NorESM2-MM, MPI-ESM1.2-HR, and CanESM5) and their ensemble average data under two Share Socioeconomic Pathways (i.e., SSP2-4.5 and SSP5-8.5) were statistically downscaled through the SCA technique to examine the future trends of projected temperature, (iii) calculate the temperature based indices Daily Temperature Range (DTR), Frost Days (FD), Ice Days (ID), Summer Days (SU), Growing Degree Days (GDD), and Growing Season Length (GSL), iv) calculate the magnitudes of expected changes in the Tmax and Tmin and their relevant selected indices during observed and two future periods: FP1 (2026–2050) and FP2 (2051–2075). The results from this research can consequently help policymakers and farmers explore the possible adaptation plans against the changing climate in PEI.

## Materials and methods

### Study area

The study area chosen for this research is Prince Edward Island (PEI), the smallest province of Atlantic Canada in terms of land and population. This island is located 46–47°N and 62–64°W in the Gulf of Saint Lawrence. The island’s climate is mild, mainly influenced by the surrounding warm water of the Gulf of Lawrence. This region is highly vulnerable to climate change due to low-laying topography and extensive coastline. During summer, the temperature ranges from 20 to 34 °C, while in winter, temperatures drop to − 11 to − 3 °C^33^. July and August are PEI’s warmest and driest months^34^. It was also observed that the PEI average of daily mean temperature, mean daily minimum temperature and continuous dry days significantly increased by 0.77 °C, 1.17 °C, and 3.33 days, respectively, for the past three decades. For the same period, decreasing trends were observed of − 1.01 °C, − 3.75 days, − 5.67 days, − 11.40 nights, and − 2.00 days for daily temperature range, frost days, cold days, cold nights, and warmest day, respectively^6^. Farming is the backbone of the PEI economy, with nearly half of its total land area, 240,514 out of 566,560 hectares, used for agriculture^35^. The agriculture sector of PEI also faces numerous challenges due to climate change, mainly because a significant portion of the agricultural land relies on rainfall for irrigation^6^. To assist farmers and policymakers, it is essential to project the future climate extremes of the island.

### Datasets

**Figure 1:**
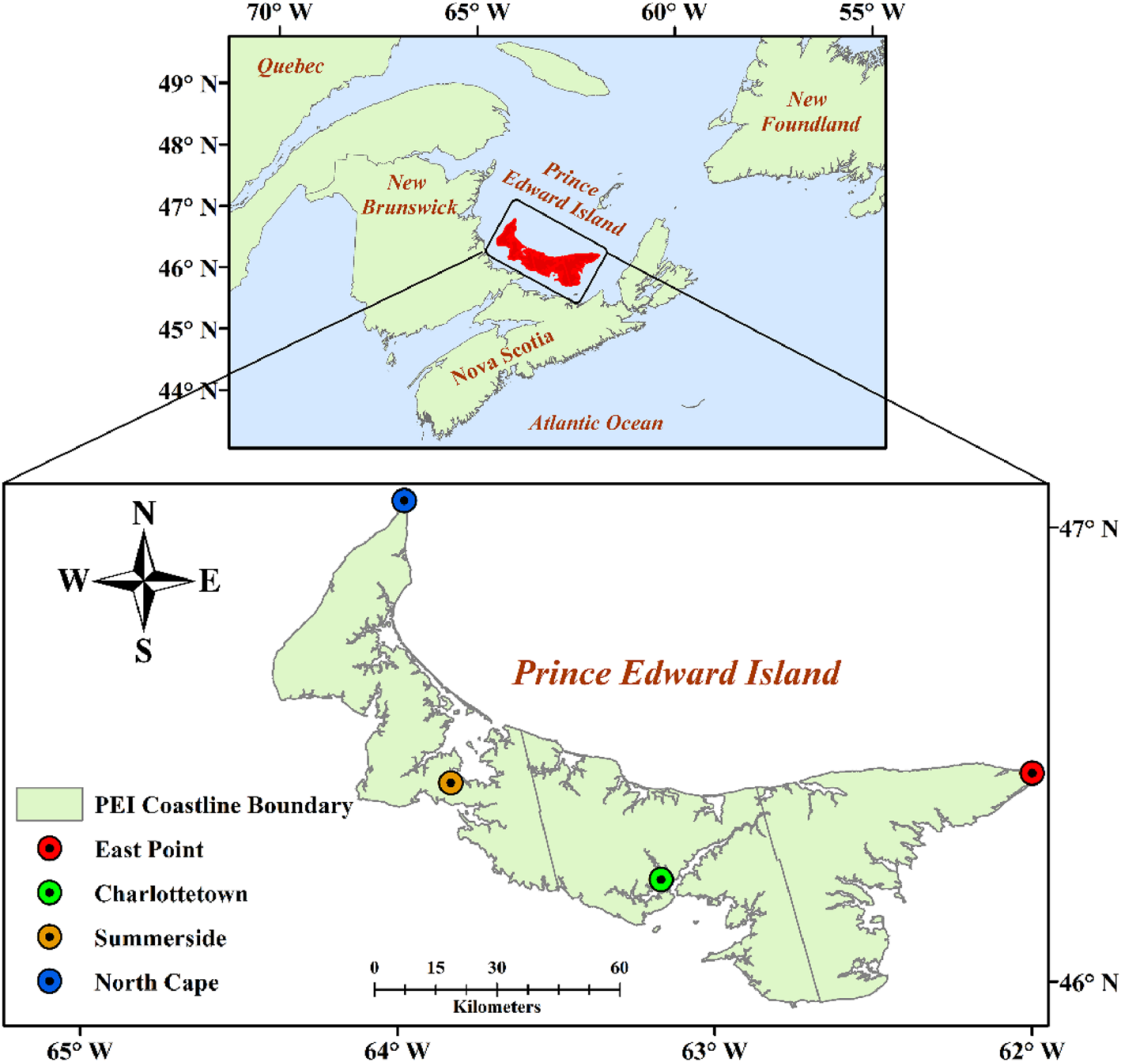
Geographical map showing the study area and four selected meteorological stations within Prince Edward Island, Canada. The map was generated using ArcMap 10.7.1 (https://www.esri.com).

Four meteorological stations, namely East Point (46.43°N, − 62.68°W), Charlottetown (46.29°N, − 63.13°W), Summerside (46.44°N, − 63.83°W), and North Cape (46.85°N, − 64.02°W), were strategically selected based on their random distribution across the island, extensive data availability, and high data quality. The daily observed data of Tmax and Tmin for the historical period (1989–2014) of four meteorological stations located throughout the PEI (Fig. 1) were acquired from Environment Canada^36^. The data was missing around 0.41% at East Point, 0.65% at Charlottetown, 9.7% at Summerside, and 1.17% at North Cape. To estimate all these missing values, the linear regression model utilized data from the neighboring meteorological station that has a high degree of similarity with the selected station’s data (R^2^ > 90.0). This approach ensured that all the estimated values aligned closely with the observed data.

In this study, two reanalysis datasets, the National Centers for Environmental Prediction-Department of Energy (NCEP-DOE) Atmospheric Model Intercomparison Project (AMIP)-II Reanalysis (also called NCEP-DOE Reanalysis 2)^37^ as well as European Centre for Medium-Range Weather Forecasts (ECMWF) Atmospheric Reanalysis Fifth Generation (ERA5)^38^, were utilized to calibrate and validate the downscaling model (Table 1). NCEP-DOE Reanalysis 2 represents an enhanced version of the NCEP analysis, which rectifies errors and incorporates updated parameterizations of physical processes. This improved version addresses the previous limitations and provides more accurate and reliable results^39^. ERA5, a successor to ERA-Interim reanalysis, employs advanced modeling and data assimilation systems to merge extensive historical observations into comprehensive global estimates^40^. These reanalysis datasets were chosen over the predictors of the Global Circulation Models (GCMs) due to their high level of correctness and accuracy. Three GCMs from the Coupled Model Intercomparison Project Phase 6 (CMIP6) were employed in this study (Table 1) to examine the future variations (2015–2100) of Tmax and Tmin under two Shared Socioeconomic Pathways (SSP2-4.5 and SSP5-8.5). These GCMs include Norwegian Earth System Model version 2 (NorESM2-MM), Max Planck Institute for Meteorology Earth System Model version 1.2 (MPI-ESM1.2-HR), and Canadian Earth System Model version 5 (CanESM5) (Table 1). These GCMs were selected based on their different Equilibrium Climate sensitivity (ECS) values. The NorESM2-MM possesses a low ECS, MPI-ESM1.2-HR exhibits medium ECS, and CanESM5 has a high ECS value (Table 1). All these GCMs are the current and updated versions like NorESM2-MM is the latest version of NorESM1, MPI-ESM1.2-HR is an updated version of MPI-ESM, and CanESM5 is the current version of CanESM2. Hence, the most recent and updated reanalysis datasets and GCMs were utilized in this study to minimize any error or uncertainty. The future projection considers different Shared Socioeconomic Pathways (SSPs), including SSP1-2.6, SSP2-4.5, SSP3-7.0, and SSP5-8.5, as defined by CMIP6. However, the current study focuses solely on two SSPs: SSP2-4.5, which represents the middle-of-the-road scenario, and SSP5-8.5, which depicts the fossil-fueled development pathways. These selected emission scenarios align with the corresponding global Representative Concentration Pathways^7^. The r1i1p1f1 variant of each GCM was used in this study. Both reanalysis and GCMs datasets were acquired from the Canadian climate data and scenarios website^41^, which provides a grid-based dataset. The whole island is encompassed by two grids (106X_49Y and 107X_49Y), each has a native CanESM5 grid resolution of 2.8125° × 2.8125° (312.47 × 312.47 km). To ensure consistency and better comparison, all other datasets (reanalysis and GCMs) were interpolated using the Spherical Harmonics Interpolation method to match the CanESM2 grid resolution. This interpolation method transforms data from a fixed grid to a Gaussian grid on the sphere’s surface. The f2gsh_Wrap (fixed to Gaussian grid spherical harmonic wrapper) tool within NCL (NCAR Command Language) was used to perform this interpolation.

**Table 1:** List of Global Circulation Models (GCMs) and reanalysis datasets, their institute, resolution, Equilibrium Climate Sensitivity (ECS) values, variant label, Shared Socioeconomic Pathways (SSPs), and period used in this study.

Data	Reanalysis dataset & model	Institute	ECS (°K)	Resolution	Variant label	SSPs	Period
Reanalysis dataset	NCEP-DOE Reanalysis 2	National Centre for Environmental Prediction	N/a	312.47 × 312.47 km	N/A	N/A	1989–2014
	ECMWF ERA5	European Centre for Medium-Range Weather Forecasts	N/a	312.47 × 312.47 km	N/A	N/A	1989–2014
Global circulation model	NorESM2-MM	Norwegian Earth System Model	2.5	312.47 × 312.47 km	r1i1p1f1	SSP2-4.5, SSP5-8.5	2015–2100
	MPI-ESM1.2-HR	Max Planck Institute Earth System Model	3.0	312.47 × 312.47 km	r1i1p1f1	SSP2-4.5, SSP5-8.5	2015–2100
	CanESM5	Canadian Earth System Model version 5	5.6	312.47 × 312.47 km	r1i1p1f1	SSP2-4.5, SSP5-8.5	2015–2100

Reanalysis datasets do not have a variant ID and ECS value and are available only for historical periods; therefore, multiple columns are marked as not applicable (N/A).

## Methodology

The Stepwise Cluster Analysis (SCA) technique was employed in this study to downscale and future project the Tmax and Tmin for four selected meteorological stations in PEI. The research methodology involved the following steps: (i) screening of the predictors, (ii) comparison of reanalysis datasets and model validation, (iii) bias correction, (iv) projection of Tmax and Tmin, (v) Calculate temperature extreme indices and their trends for the baseline period (1989–2014) and two future periods: FP1 (2026–2050) and FP2 (2051–2075).

### Screening of predictors

The screening of reanalysis predictors plays a vital role in statistical downscaling methods, as these carefully selected predictors significantly impact the output of the models. This step holds utmost importance and is considered crucial in the overall process. First, the reanalysis dataset was extracted for the same period as the observed dataset (1989–2014) for a better correlation between these datasets. Then, predictors were assessed by following the steps used by Maqsood et al.^42^, where a combination of statistical measures correlation, partial correlation, and *p*-value were used to evaluate the relationship between the observed data and reanalysis predictors. These steps help to identify the most relevant predictors of the targeted variables. This screening approach aimed to enhance the accuracy and reliability of the downscaling results by focusing on the predictors that demonstrated the strongest correlations and statistical significance.

### Stepwise cluster analysis

Stepwise Cluster Analysis (SCA) is employed in this study to downscale the Tmax and Tmin. It is an R-package developed by Wang et al.^28^ and used to perform this modeling. Being a multivariate statistical model, SCA creates a statistical relationship between local surface variables (predictands) and large-scale atmospheric variables (predictors) by grouping pairs of predictors and predictands into clusters without assuming functional relationships. This grouping process involves iterative cutting and merging actions, where clusters are initially separated into smaller clusters, and then similar small clusters are merged. So, the core principle of SCA is based on a series of cutting and merging operations to divide clusters (sample sets) containing multiple related and independent variables into indivisible subclusters^28^. The SCA algorithm is based on the multivariate analysis of variance (MANOVA) theory, which helps identify differences between two sets of dependent variables. The process involves dividing the original dependent variable set into unrelated subsets based on specific criteria. A cluster tree is generated by rejecting further cutting or merging hypotheses (final model), which represents the complex relationship (e.g., non-functional, nonlinear) between independent and dependent variables. The values of independent variables serve as references to determine the cluster/leaf that a sample from the original set will be assigned. Using the obtained clustering tree, predictions can be made for new input data to estimate the corresponding output. Thus, the SCA is based on a classification approach, where the Wilks statistic and F-test serve as classification criteria. Instead of relying on a specific function, the cluster tree reflects the relationship between climate outputs and actual observations.

### Bias correction

Certain biases exist in the model’s output, which can be addressed using bias correction methods. These uncertainties arise in the results of models due to insufficient calibration, lack of observed data, and the complexity of the climatic system Balov and Altunkaynak^43^. Bias correction methods are employed to remove the biases and improve the accuracy of the model’s generated results for the historical and future periods. These methods estimate the bias factor by comparing the observed and model-simulated results of the baseline period and then utilize that factor to remove the biases in model-simulated and future projected data. This study utilized the quantile delta mapping (QDM) method to remove the biases. This method maintains the quantile changes and is equivalent to the equidistant and equation forms of quantile mapping. The model projection undergoes quantile detrending initially, and the simulated value is then bias-corrected using quantile mapping. The transfer function for this correction is constructed during the calibration period. Finally, the projected absolute changes (for temperature) in quantiles are added to the bias-corrected model outputs to yield the final results.

The calculation is based on the Python language package ‘cmethods’, https://github.com/btschwertfeger/python-cmethods.git, which implemented the equations of Tong et al.^31^.

### Temperature extreme indices and their trends analysis

Six temperature extreme indices defined by the Expert Team on Climate Change Detection and Indices (ETCCDI) were calculated for the baseline and two future periods (FP1 and FP2). The details of these selected indices are given in Table 2. These calculations were performed using ClimPACT2 software developed by Alexander and Herold^44^. This software incorporates several data quality control processes to ensure the accuracy and reliability of the calculated indices^4^. The minimum errors were found in the data and eliminated before computing the indices by taking appropriate steps.

**Table 2:** Description of temperature extreme indices used in this study.

Index ID	Index name	Definition	Unit
SU	Summer days	Number of days when Tmax > 25 °C	Days
ID	Ice days	Number of days when Tmax < 0 °C	Days
FD	Frost days	Number of days when Tmin < 0 °C	Days
DTR	Daily temperature range	Tmax–Tmin	°C
GSL	Growing season length	Annual number of days between the first occurrence of 6 consecutive days with Tmean > 5 °C and the first occurrence of 6 consecutive days with Tmean < 5 °C	Days
GDD	Growing degree days	A measure of heat accumulation to predict plant and animal developmental rates	Degree/days

*Tmax* Daily maximum temperature, *Tmin* Daily minimum temperature, *Tmean* Daily minimum temperature.

The selected six temperature indices are crucial in agricultural decision-making, crop selection, and resource management. The Growing Degree Days (GDD) and Growing Season Length (GSL) indices are commonly used in agriculture and climatology to assess the suitability of a region for various crops and to understand the length of the growing season. GDD measures accumulated heat units over a specific period, usually from the beginning of the growing season to a specific threshold temperature^45^. GSL determines the length of time available for planting, growth, and harvesting of crops. Frost days (FD) is a crucial index for understanding the potential risks and planning necessary measures to protect crops from cold temperatures. Frost days can damage crops, which results in a reduction in agricultural production^46^. Daily temperature range (DTR) provides insights into temperature fluctuations and variations that crops may experience during their growth cycle^47^. Ice days (ID) are decreasing, while summer days (SU) are increasing throughout Canada^48^. These indices can also provide valuable guidance in planting and harvesting crops.

The Modifiedmk R-package was used to calculate trends in selected indices^49^. This package is a modified version of the Man-Kendall test designed to analyze the non-random data influenced by autocorrelation. This test first examines the data for autocorrelation; if present, it employs a prewhitening technique proposed by Hamed^50^. This technique simultaneously estimates the slope and lag-1 serial correlation coefficient, correcting the latter for bias before prewhitening. After prewhitening, the test utilized the Man-Kendall test to identify increasing or decreasing trends and calculates changes per year using Sen’s slope test. This test determined the direction of the trends, enabling us to accurately assess changes over time and their statistical significance.

### Statistical evaluators

To evaluate the stepwise cluster analysis downscaling method and evaluate the bias correction method, four statistical parameters (R-square (R^2^), Nash–Sutcliffe model efficiency coefficient (NSE), root mean square error (RMSE), and mean absolute error (MAE)) were selected.

The R^2^ is mainly used to measure the model’s goodness-of-fit and strength of the relationship between simulated and observed values. A value close to “0” indicates a low or no relationship, while values close to “1” show a strong relationship between simulated and observed values.

$${\text{R}}^{2} = 1 - \sqrt {\frac{{\mathop \sum \nolimits_{i = 1}^{N} \left( {Y_{i}^{obs} - Y^{mean} } \right)^{2} - \mathop \sum \nolimits_{i = 1}^{N} \left( {Y_{i}^{sim} - Y_{i}^{obs} } \right)^{2} }}{{\mathop \sum \nolimits_{i = 1}^{N} \left( {Y_{i}^{obs} - Y^{mean} } \right)^{2} }}}$$

It is important to note that relying solely on the R^2^ value does not comprehensively evaluate the model’s performance. Therefore, this study also incorporates other statistical indicators, namely NSE and RMSE, to achieve a more comprehensive assessment. The NSE is used to evaluate how effectively a model captures the observed variation and performs in comparison to the mean of the observed values. The NSE coefficient has a range from negative infinity to 1, and a value closure to 1 represents the best-performing model.

$${\text{NSE}} = 1 - \frac{{\mathop \sum \nolimits_{i = 1}^{N} \left( {Y_{i}^{sim} - Y_{i}^{obs} } \right)^{2} }}{{\mathop \sum \nolimits_{i = 1}^{N} \left( {Y_{i}^{obs} - Y^{mean} } \right)^{2} }}$$

The RMSE is an error index that measures the average magnitude of the error between simulated and observed values. A smaller RMSE value closure to “0” indicates the best performance of the model.

$${\text{RMSE}} = \sqrt {\frac{{\mathop \sum \nolimits_{i = 1}^{N} \left( {Y_{i}^{sim} - Y_{i}^{obs} } \right)^{2} }}{N}}$$

The MAE tells how big an error we can expect from the measurement by averaging the amount of errors present in the measurements.

$${\text{MAE}} = \frac{1}{N}\mathop \sum \limits_{i = 1}^{N} \left( {Y_{i}^{sim} - Y_{i}^{obs} } \right)^{2}$$

where, $Y_{i}^{obs} and Y_{i}^{sim}$ are the actual and simulated values at the ith time, respectively. i ranges from 1 to N with N being the total number of values. $Y^{mean}$ represents the mean value of the ${Y}_{i}$. The performance of the reanalysis dataset was assessed based on the higher NSE and﻿ ﻿R^2^ values, and lower value of RMSE.

## Results and discussions

### Predictors screening

Initially, 26 predictors of reanalysis datasets (NCEP-DOE Reanalysis 2 and ECMWF ERA5) were used during the screening process. Then, a combination of correlation matrix, *p*-value, partial correlation, and percentage of reduction in partial correlation (PRP) was used to screen the predictors^46^. The steps are explained below.

1. First, extract the atmospheric variables for the length of the observed period (1989–2014) for better correlation.
2. Create a correlation matrix between the observed (Tmax and Tmin) and atmospheric predictors, select 12 predictors that have a high correlation (R_1) with the observed data, and arrange them in descending order.
3. The “temp” was the super predictor as it had the highest correlation (> 0.89) for both Tmax and Tmin at all the stations.

**Figure 2:**
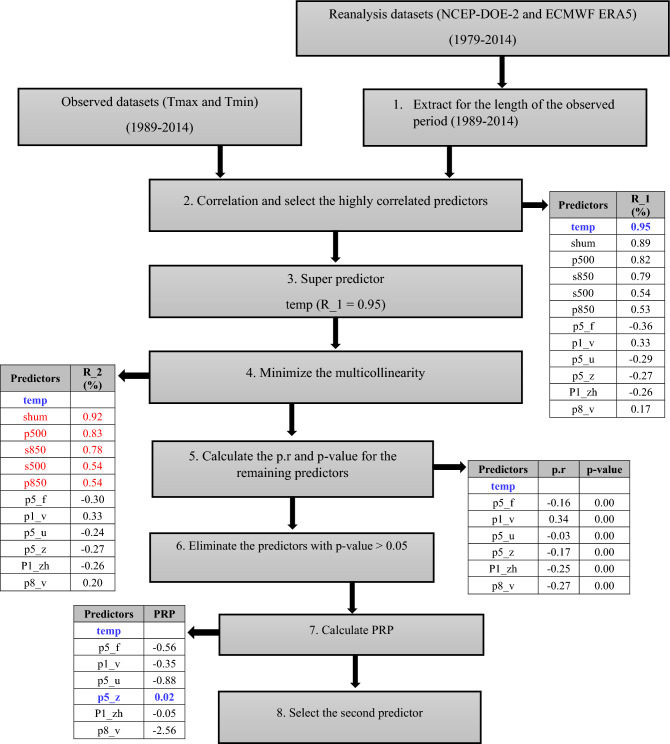
Schematic diagram for screening of predictors. In this diagram, the red font shows the eliminated predictors and the blue font shows the selected predictor.

Screening of atmospheric predictors for Tmin at North Cape station is explained below with the help of a schematic diagram (Fig. 2). The predictor “temp” was the super predictor as it has the highest correlation (R_1 = 0.95) with observed data. It was the first screened predictor. The following steps were followed to find the next predictor.

4. In the next step, find the correlation (R_2) between selected 12 predictors and eliminate those predictors that had high correlation (R_2 > 0.5) with super predictor (temp) to minimize the multicollinearity. In the following example, the red-colored predictors were eliminated as they have a high correlation (R_2 > 0.5) with the super predictor (temp).
5. Calculate the p.r and p-value for the remaining predictors (p5_f, p1_v, p5_u, p5_z, p1_zh, and p8_v).
6. Eliminate the predictors that have a *p*-value > 0.05. In the bottom example, all the predictors used in step-5 were retained to calculate the PRP, as all the predictors have p-values less than 0.05.
7. The percentage of reduction in partial correlation to the correlation coefficient (PRP) was used to find the second most appropriate atmospheric predictor.
  $${\text{PRP }} = \frac{{{\text{p}} \cdot {\text{r}} - {\text{R}}\_1}}{{{\text{R}}\_1}}$$
8. The predictor with low PRP was selected as a second predictor.
  P5_z was selected as it had the lowest PRP value (0.02) among all other predictors.

For the Tmax variable, the temp (mean temperature at 2 m) and p500 (500 hpa geopotential height) predictors from both reanalysis datasets (NCEP-DOE Reanalysis 2 and ECMWF ERA5) underwent screening. In the case of Tmin, the temp (mean temperature at 2 m) and p5z (500 hpa vorticity) predictors were selected. The temp predictor was observed as the super predictor for both Tmax and Tmin variables. These findings align with similar observations made in other studies, which further reinforce the significance of these super predictors in temperature modeling^42,51^.

### Model’s evaluation and comparison of reanalysis datasets

**Figure 3:**
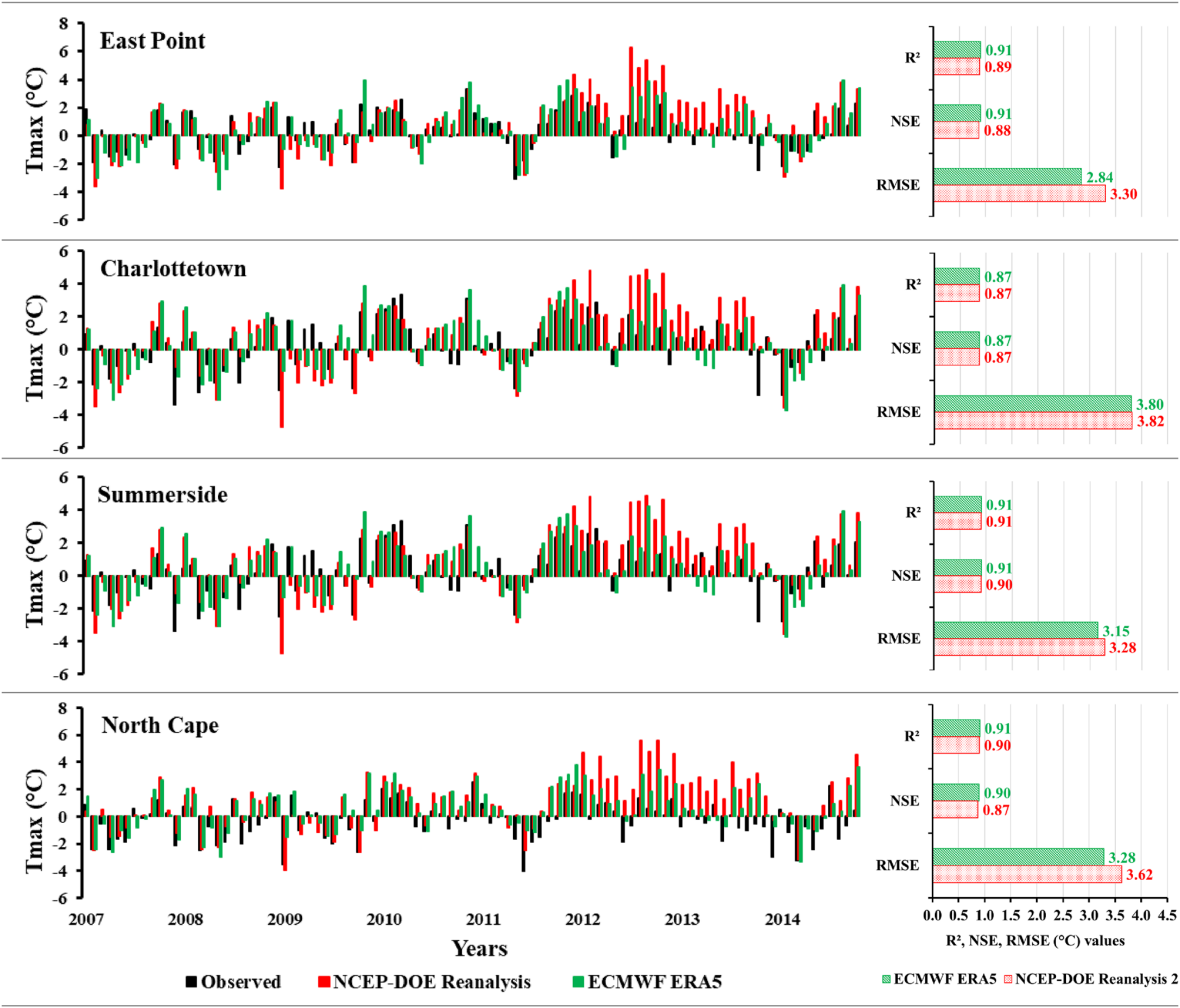
Monthly average anomalies of the daily maximum temperature (Tmax) using two reanalysis datasets (NCEP-DOE Reanalysis 2 and ECMWF ERA5) during the validation period (2007–2014) with respect to the baseline period (1989–2014) and statistical parameters to evaluate the models.

**Figure 4:**
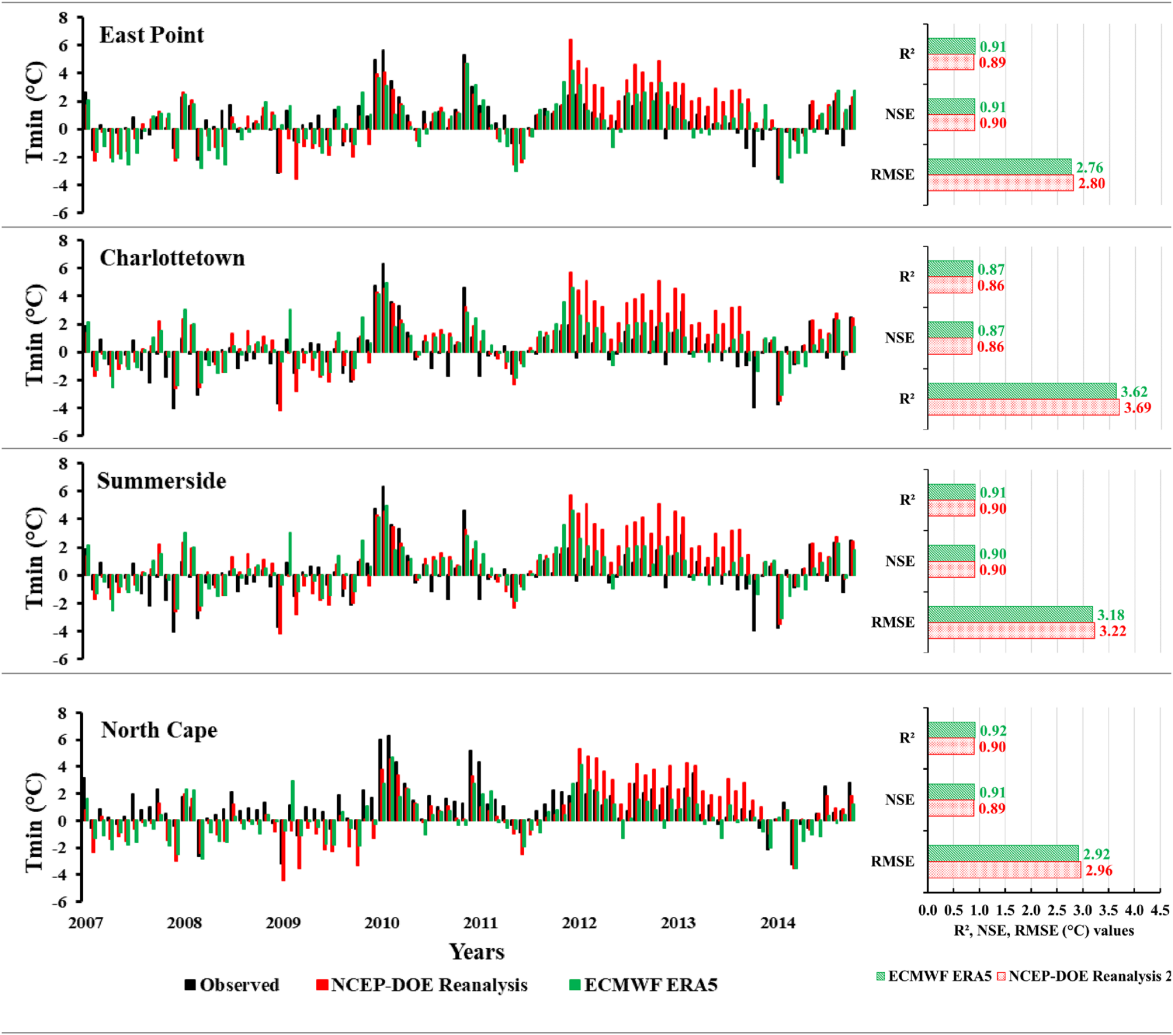
Monthly average anomalies of the daily minimum temperature (Tmin) using two reanalysis datasets (NCEP-DOE Reanalysis 2 and ECMWF ERA5) during the validation period (2007–2014) with respect to the baseline period (1989–2014) and statistical parameters to evaluate the models.

To evaluate the SCA model, the baseline period (1989–2014) was divided into 70% (1989–2006) for calibration and 30% (2007–2014) for validation. The screened predictors were employed during the calibration and validation period. The performance of the SCA model in downscaling Tmax and Tmin was found to be satisfactory. The simulated results demonstrated a strong correlation (R^2^ > 0.86) with the observed data at all four stations for both reanalysis datasets (Figs. 3 and 4). Lu et al.^52^ also utilized the SCA model to downscale Tmax and Tmin and reported similarly favorable results. Their study revealed a strong performance of the SCA model (R^2^ > 0.84) in downscaling these climatic predictors. The downscaling performance of the ECMWF ERA5 and NCEP-DOE Reanalysis 2 datasets for Tmax and Tmin was also accessed. The R^2^ values are almost the same at all the stations for both reanalysis datasets. However, the ECMWF ERA5 performed better than the NCEP-DOE Reanalysis 2 in terms of NSE and RMSE values. The NSE values for ECMWF were slightly higher at most stations and had fewer errors (RMSE) at all the stations from 2.84 to 3.80 °C compared to the NCEP-DOE Reanalysis 2, which has an error range from 3.28 to 3.82 °C (Fig. 3). The monthly anomalies using NCEP-DOE Reanalysis 2 datasets overestimate the Tmax most of the time, while the anomalies using ECMWF ERA5 simulated time series for both Tmax and Tmin have better agreement with the observed data, as illustrated in Figs. 3 and 4.

For Tmin, the R^2^ and NSE values were slightly higher (0.1–0.2) for ECMWF ERA5 as compared to the NCEP-DOE Reanalysis 2. Additionally, NCEP-DOE Reanalysis 2 had higher errors ranging from 2.80 to 3.69 °C, while ECMWF ERA5 had errors ranging from 2.76 to 3.62 °C (Fig. 4). De Lima et al.^53^ also compared the previous versions of these reanalysis datasets (ERA/Interim ECMWF, NCEP/NCAR, and CFSR) to downscale the Tmax and Tmin and observed better results for ERA/Interim ECMWF and found more errors for NCEP/NCAR. The comparison of reanalysis datasets in downscaling the Tmax and Tmin indicates that ECMWF ERA5 performed slightly better than NCEP-DOE Reanalysis 2 as it includes the latest features and systems, has a high temporal resolution, and more consistent sea surface temperature^41^. Therefore, ECMWF ERA5 was further used to downscale the historical and future projects of the Tmax and Tmin.

**Figure 5:**
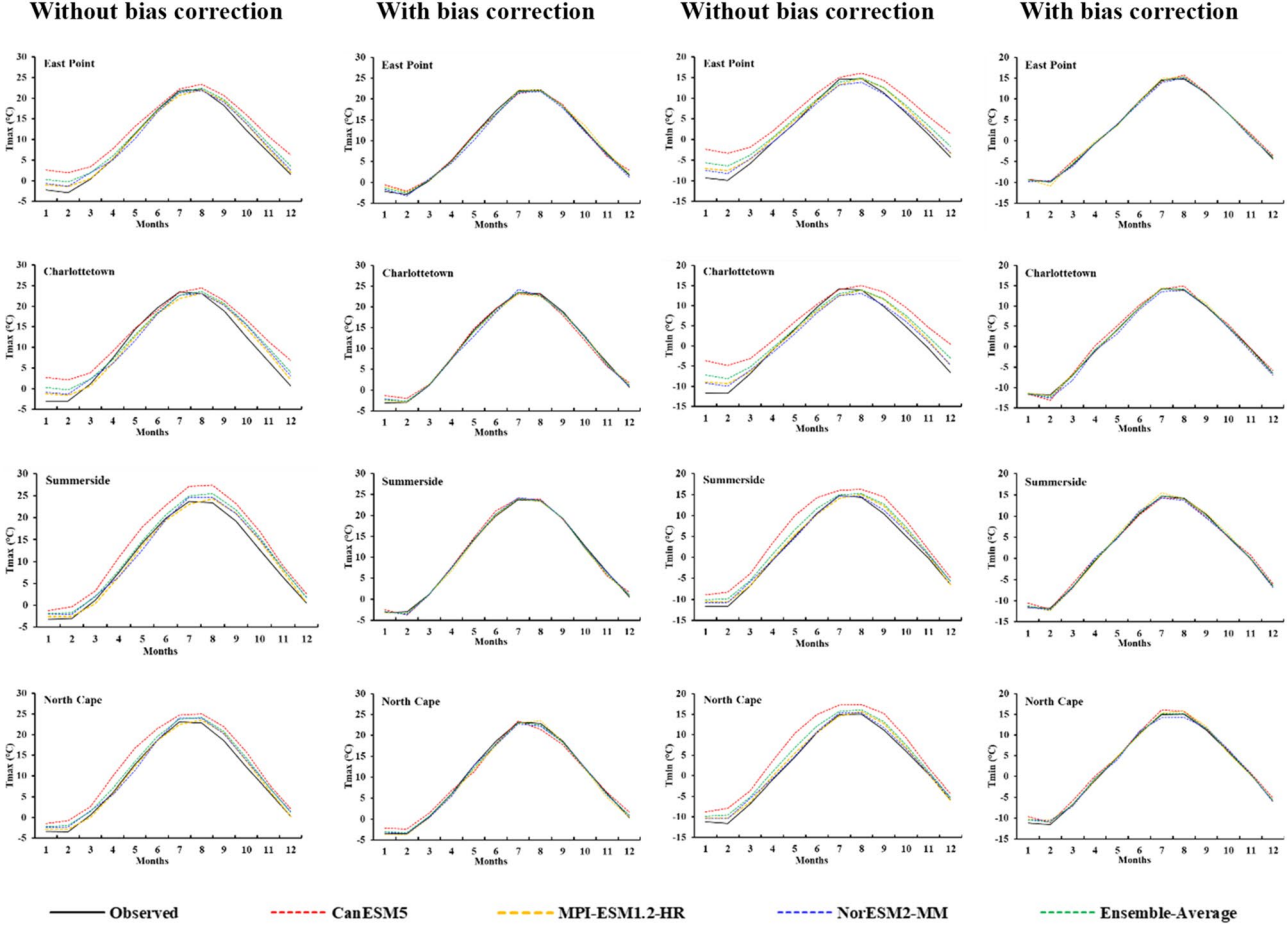
Historical performance of the NorESM2-MM, MPI-ESM-1.2-HR, CanESM5, and Ensemble-Average, with and without bias correction, to downscale the maximum temperature (Tmax (°C)) and minimum temperature (Tmin (°C)) using ECMWF ERA5-based model at East Point, Charlottetown, Summerside, and North Cape.

The ECMWF ERA5-based model was utilized first to downscale the Tmax and Tmin for the historical period using three CMIP6 GCMs (NorESM2-MM, MPI-ESM-1.2-HR, and CanESM5) separately to check their historical performance. Both the Tmax and Tmin results of the CanESM5 were not satisfactory as they showed more inconsistency (R^2^ varied from 0.70 to 0.85, with high error values RMSE 3.69–4.98﻿ °C and MAE 3.05–3.96 ﻿°C) with the observed data compared to the NorESM2-MM and MPI-ESM-1.2-HR (Table 3). Due to its high ECS value, it overestimated the Tmax and Tmin at all the stations (Fig. 5). The QDM method was utilized to remove these biases from the GCM-simulated Tmax and Tmin at all the stations for the historical period. The QDM method performs well in removing biases as after the bias correction, the correlation of GCM-simulated Tmax and Tmin increased from 0.92 to 0.95 at all the stations. The QDM method also improved the other statistical parameter values for the simulated results of Tmax and Tmin, such as RMSE values falling between 2.11 and 2.60 ﻿°C and MAE values from 1.60 to 1.93 ﻿°C (Table 3). This bias correction method mostly improves the CanESM5 results as it had more inconsistency with the observed data. While others already had a better correlation with the observed, so it improved a little for them (Fig. 5) (Table 3). Overall, the QDM method resulted in satisfactory results, reducing the biases between observed and simulated daily Tmax and Tmin.

**Table 3:** Comparison of without and quantile delta mapping bias-corrected climate model simulations.

Stations		GCMs	Without bias correction			With bias correction		
			R^2^	RMSE	MAE	R^2^	RMSE	MAE
East Point	Tmax	CanESM5	0.81	3.90	3.23	0.93	2.33	1.82
		MPI-ESM-1.2-HR	0.92	2.36	1.88	0.94	2.26	1.77
		NorESM2-MM	0.92	2.39	1.86	0.94	2.18	1.72
	Tmin	CanESM5	0.72	4.61	3.74	0.94	2.11	1.60
		MPI-ESM-1.2-HR	0.91	2.48	1.86	0.94	2.16	1.64
		NorESM2-MM	0.92	2.35	1.77	0.94	2.14	1.62
Charlottetown	Tmax	CanESM5	0.79	4.29	3.42	0.93	2.45	1.93
		MPI-ESM-1.2-HR	0.91	2.76	2.2	0.94	2.33	1.76
		NorESM2-MM	0.90	2.94	2.25	0.94	2.30	1.78
	Tmin	CanESM5	0.70	4.98	3.96	0.92	2.18	1.66
		MPI-ESM-1.2-HR	0.91	2.70	2.06	0.95	2.12	1.64
		NorESM2-MM	0.91	2.62	2.00	0.93	2.26	1.71
Summerside	Tmax	CanESM5	0.83	4.02	3.45	0.93	2.54	1.96
		MPI-ESM-1.2-HR	0.93	2.45	1.95	0.95	2.24	1.74
		NorESM2-MM	0.93	2.64	2.08	0.95	2.26	1.75
	Tmin	CanESM5	0.82	3.99	3.33	0.93	2.41	1.79
		MPI-ESM-1.2-HR	0.93	2.40	1.87	0.94	2.26	1.79
		NorESM2-MM	0.94	2.30	1.75	0.95	2.23	1.73
North Cape	Tmax	CanESM5	0.85	3.69	3.05	0.93	2.60	1.98
		MPI-ESM-1.2-HR	0.93	2.38	1.92	0.94	2.32	1.86
		NorESM2-MM	0.93	2.52	1.97	0.94	2.27	1.78
	Tmin	CanESM5	0.80	4.23	3.59	0.93	2.51	1.93
		MPI-ESM-1.2-HR	0.93	2.41	1.87	0.94	2.33	1.80
		NorESM2-MM	0.93	2.33	1.77	0.94	2.32	1.77

*Tmax* Daily maximum temperature, *Tmin* Daily minimum temperature.

### Future projection and ensembling of global circulation models

**Figure 6:**
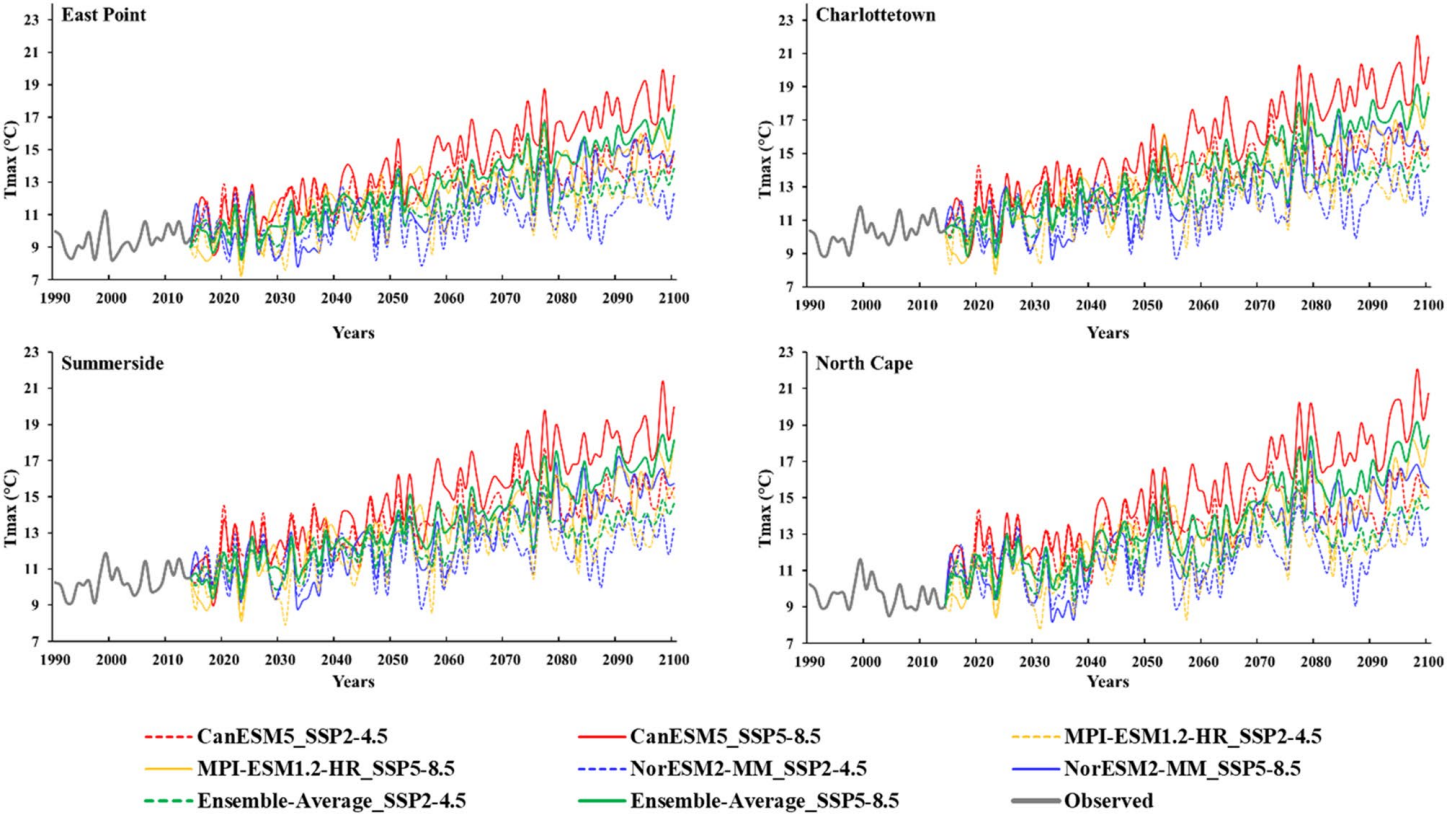
Projected changes in the bias-corrected average annual maximum temperature (Tmax (°C)) using NorESM2-MM, MPI-ESM-1.2-HR, CanESM5, and Ensemble average of selected three global circulation models at (**a**) East Point, (**b**) Charlottetown, (**c**) Summerside, and (**d**) North Cape.

**Figure 7:**
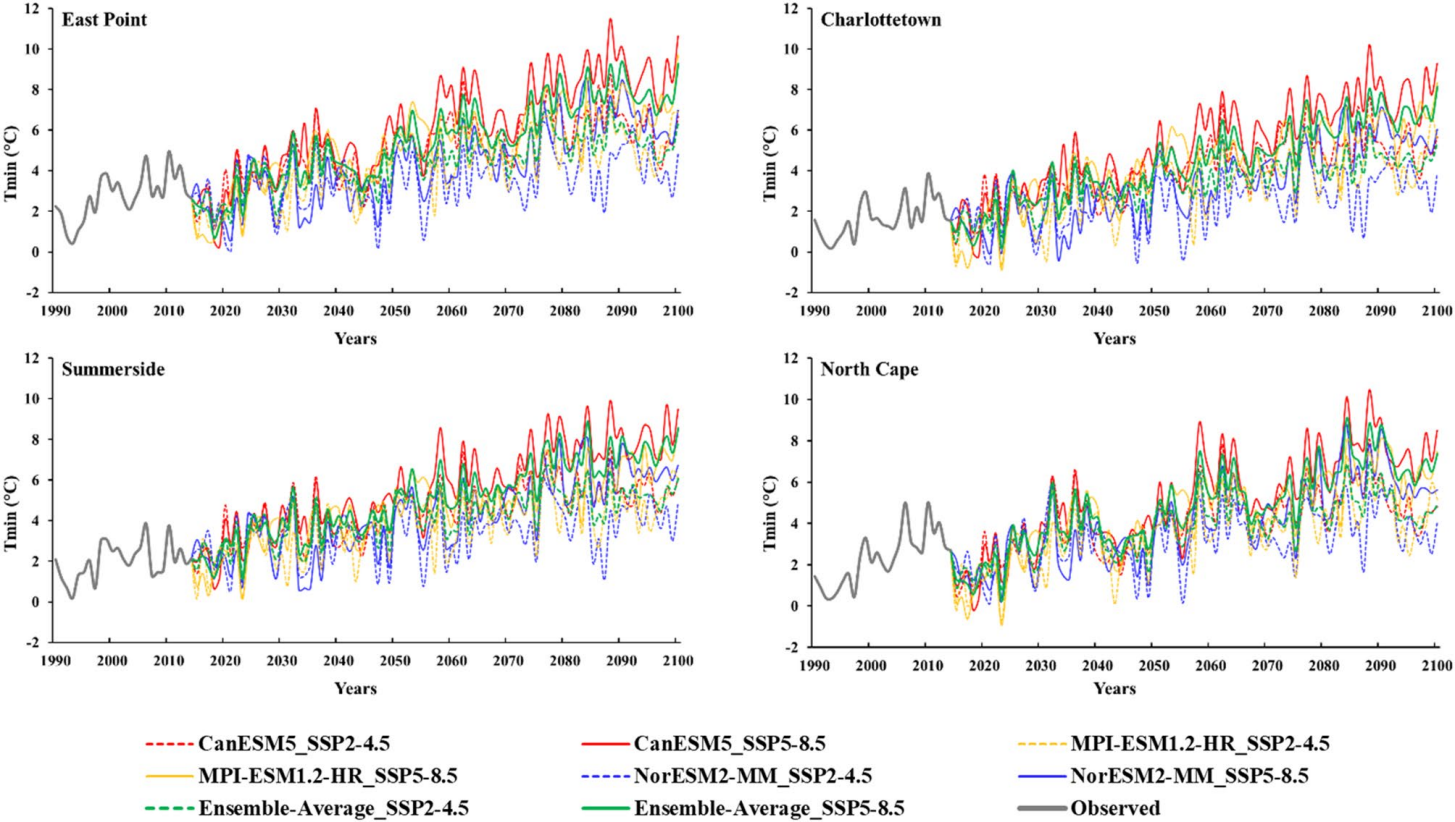
Projected changes in the bias-corrected average annual minimum temperature ((Tmin(°C)) using NorESM2-MM, MPI-ESM-1.2-HR, CanESM5, and Ensemble average of selected three global circulation models at (**a**) East Point, (**b**) Charlottetown, (**c**) Summerside, and (**d**) North Cape.

The screened ECMWF ERA5 predictors were utilized in the SCA model to perform downscaling and projections of the Tmax and Tmin using three CMIP6 GCMs (NorESM2-MM, MPI-ESM-1.2-HR, and CanESM5) separately and by taking the ensemble average of these GCMs at all the selected stations. These results were tuned first by utilizing the already built QDM method for each station, which helps to remove any biases present in the projected results (Figs. 6 and 7). Our analysis focused on two specific Shared Socioeconomic Pathways (SSPs): SSP2-4.5 and SSP5-8.5, representing different future scenarios. The future projections for Tmax exhibit variations across different models. Specifically, for the NorESM2-MM model, the average annual Tmax is expected to vary from 7.86 to 15.62 °C under SSP2-4.5 and 7.91 to 17.58 °C under SSP5-8.5 at all the stations. For the MPI-ESM-1.2-HR model, the projected average Tmax ranges from 7.19 to 15.81 °C under SSP2-4.5 and 7.32 to 18.69 °C under SSP5-8.5. The CanESM5 model suggests an average annual Tmax range of 8.55 to 18.08 °C under SSP2-4.5 and 9.33 to 22.08 °C under SSP5-8.5 at all the selected stations. Lastly, when considering the ensemble average of these GCMs, the projected average annual Tmax ranges from 15.09 to 16.20 °C under SSP2-4.5 and from 17.45 to 19.14 °C under SSP5-8.5 at all the selected stations. The projected average annual Tmax values vary slightly across different stations (Fig. 6). So, the projected average annual Tmax using the ensemble average of these GCMs at East Point ranges from 8.90 to 15.09 °C under SSP2-4.5 and 8.24 to 17.45 °C under SSP5-8.5. In Charlottetown, the projected average annual Tmax ranges from 9.56 to 16.20 °C under SSP2-4.5 and 8.78 to 19.15 °C under SSP5-8.5. Moving to Summerside, the projected average annual Tmax ranges from 9.77 to 15.66 °C under SSP2-4.5 and 9.36 to 18.44 °C under SSP5-8.5. Finally, at North Cape, the projected average annual Tmax ranges from 9.33 to 16.02 °C under SSP2-4.5 and from 9.42 to 19.17 °C under SSP5-8.5 (Fig. 6). The maximum values were observed under SSP5-8.5.

Like Tmax, the future projections for average annual Tmin also display variations, particularly maximum variation under SSP5-8.5 as depicted in Fig. 7. The future projections for average annual Tmin also exhibit variations across different models. The average annual Tmin is expected to vary from − 0.36–8.50 °C for the NorESM2-MM model, − 0.89–9.70 °C for the MPI-ESM-1.2-HR model, maximum variation − 0.21–11.48 °C was observed for CanESM5 model, and for ensemble average of these GCMs, the projected average Tmin ranges from 0.18 to 9.40 °C under SSP5-8.5 at all the selected stations. The projected Tmin using the ensemble average of these GCMs at East Point ranges from 0.70 to 9.40 °C, 0.18 to 8.14 °C at Charlottetown, 1.10–8.88 °C at Summerside, and 0.23–9.11 °C at North Cape under SSP5-8.5 (Fig. 7). The multi-model ensemble (MME) approach serves as a valuable strategy to address uncertainties among global circulation models (GCMs), enabling improved forecasting capabilities. By combining the MME approach with downscaling techniques, forecasting skills have been enhanced^54^. The simulated results for Tmax and Tmin showed that the GCM-specific changes in the ECS also impact the regional scale.

### Future projection of temperature extreme indices

**Figure 8:**
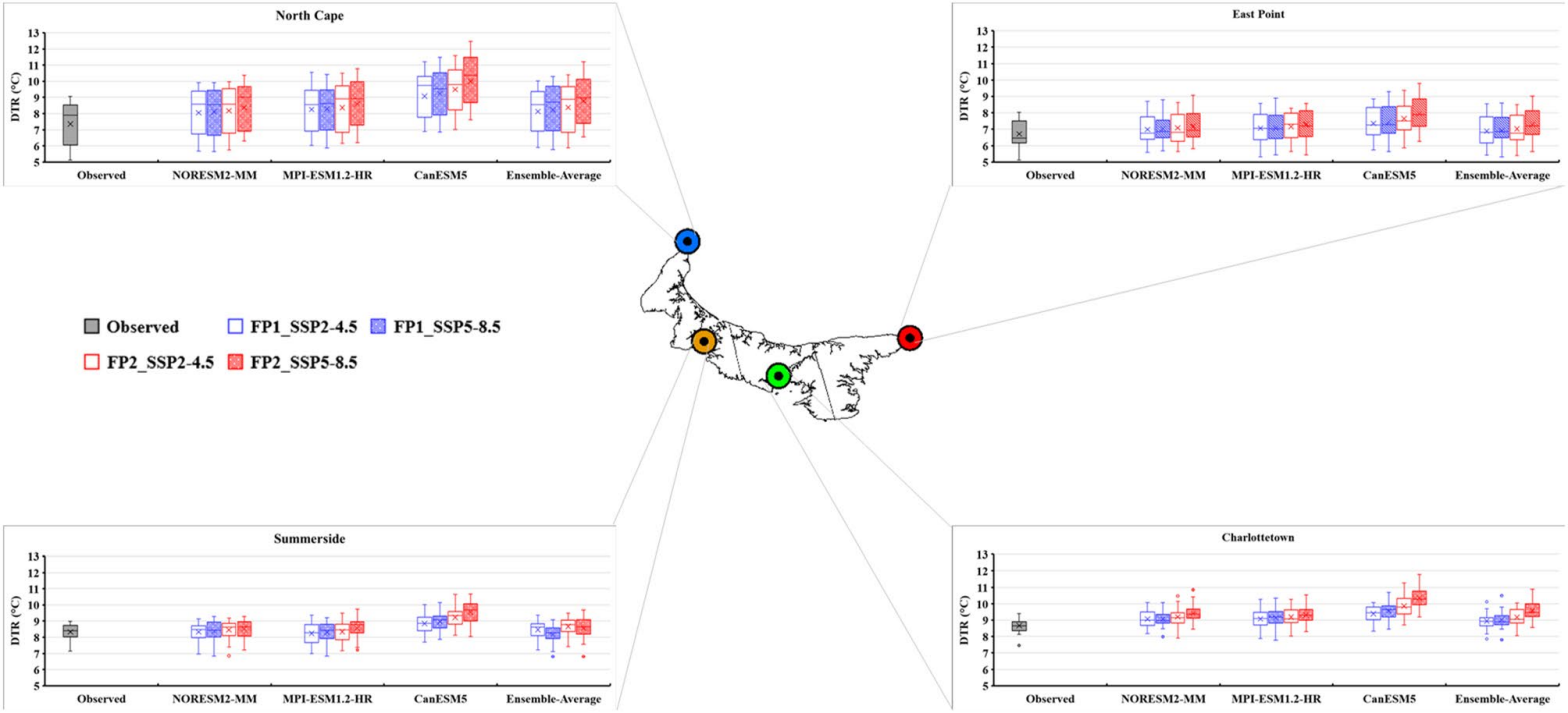
Projected variations in the average annual Daily Temperature Range (°C) for baseline (1989–2014) and two future periods: FP1 (2026–2050), FP2 (2051–2075) at four selected stations (East Point, Charlottetown, Summerside, and North Cape) under SSP2-4.5 and SPP5-8.5 scenarios. (*) asterisk sign indicates the outlier present in the data.

**Figure 9:**
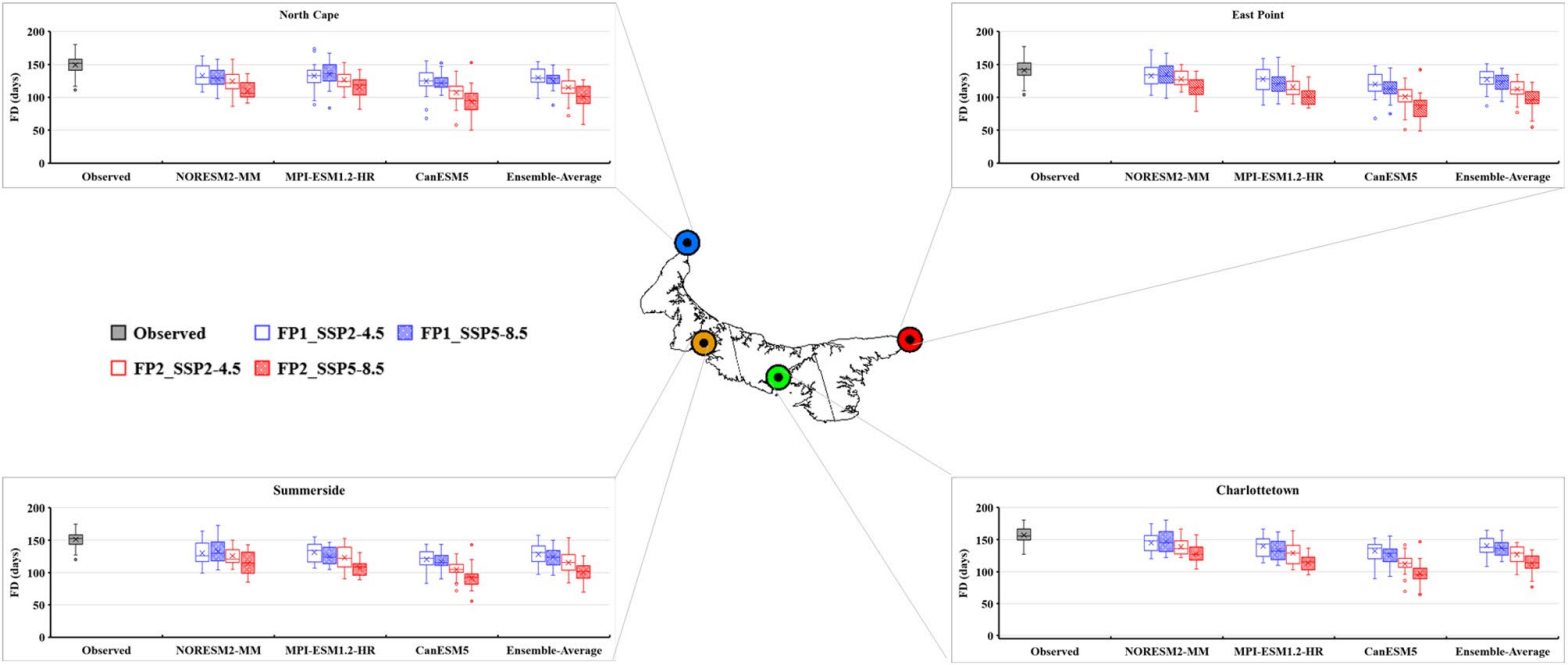
Projected variations in the average annual Frost Days (days) for baseline (1989–2014) and two future periods; FP1 (2026–2050), FP2 (2051–2075) at four selected stations (East Point, Charlottetown, Summerside, and North Cape) under SSP2-4.5 and SPP5-8.5 scenarios. (*) asterisk sign indicates the outlier present in the data.

**Figure 10:**
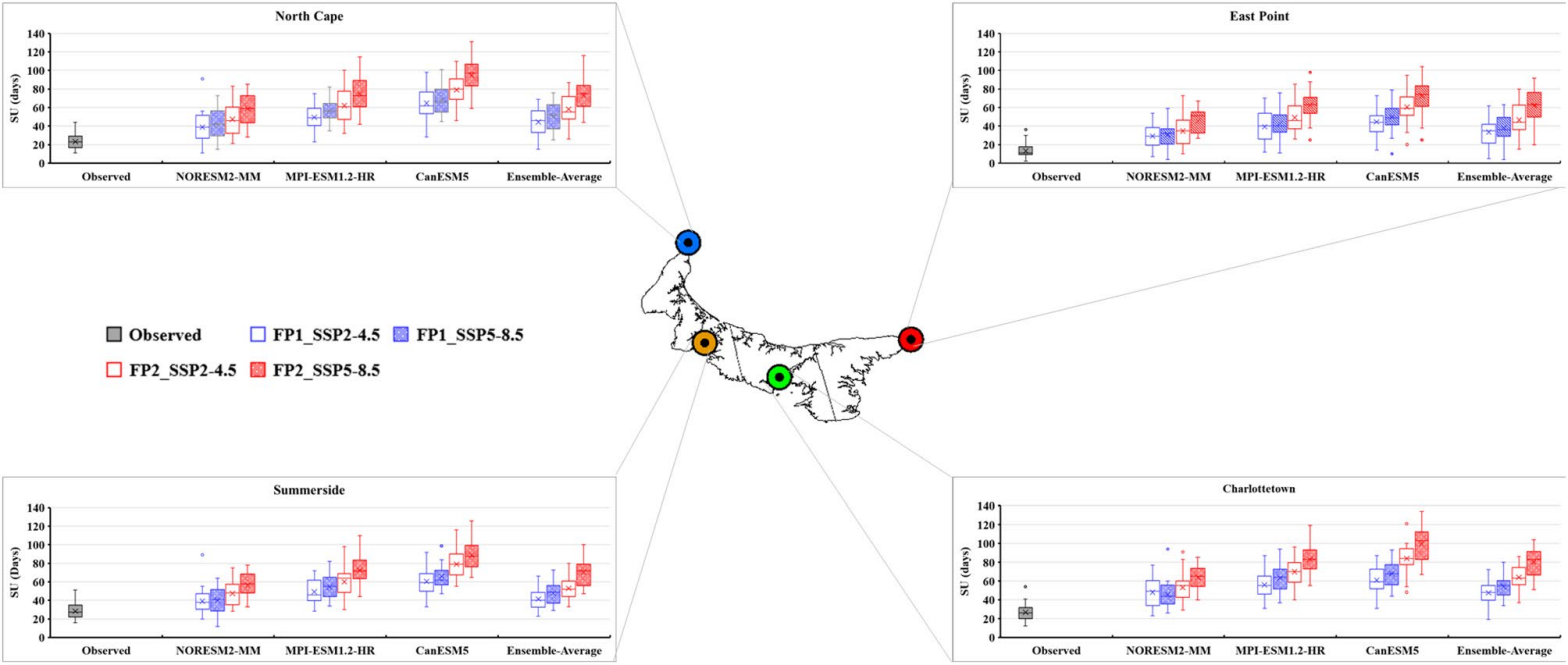
Projected variations in the average annual Summer Days (days) for baseline (1989–2014) and two future periods; FP1 (2026–2050), FP2 (2051–2075) at four selected stations (East Point, Charlottetown, Summerside, and North Cape) under SSP2-4.5 and SPP5-8.5 scenarios. (*) asterisk sign indicates the outlier present in the data.

**Figure 11:**
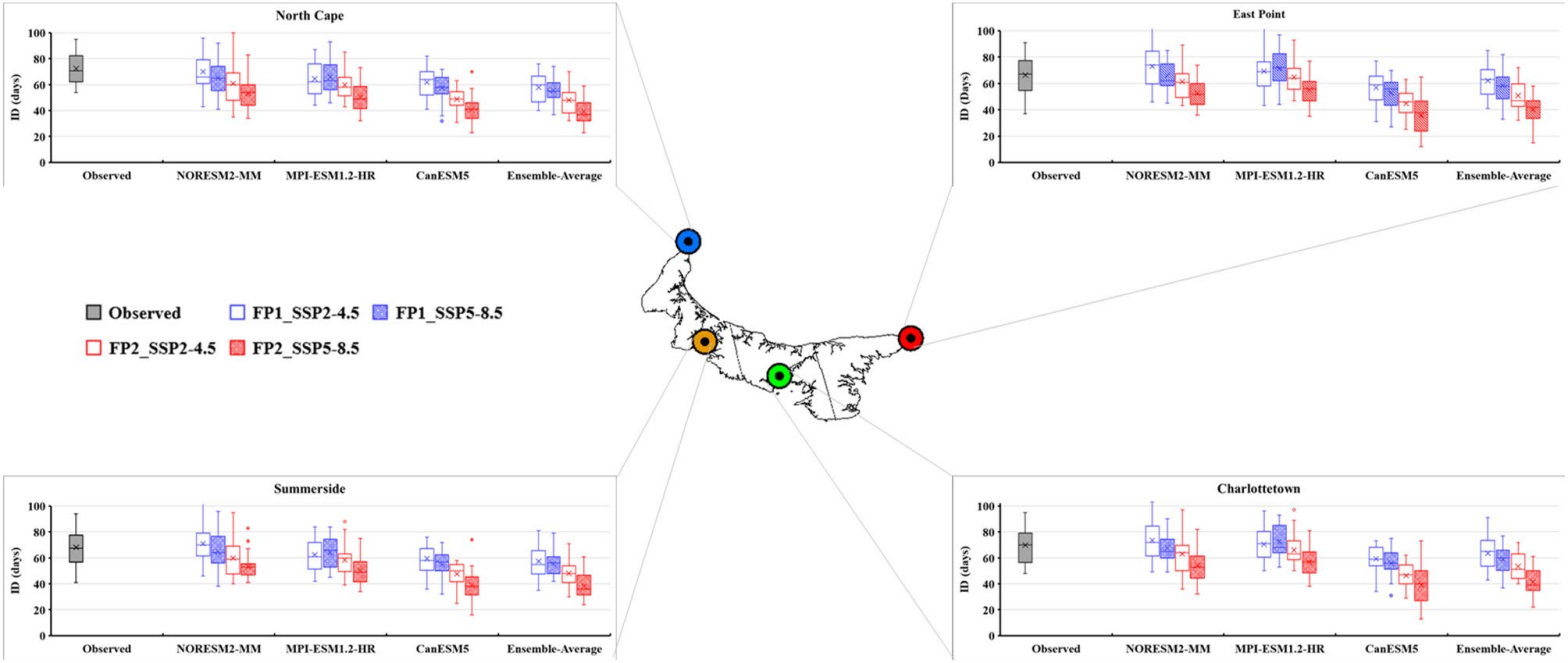
Projected variations in the average annual Ice Days (days) for baseline (1989–2014) and two future periods; FP1 (2026–2050), FP2 (2051–2075) at four selected stations (East Point, Charlottetown, Summerside, and North Cape) under SSP2-4.5 and SPP5-8.5 scenarios. (*) asterisk sign indicates the outlier present in the data.

**Figure 12:**
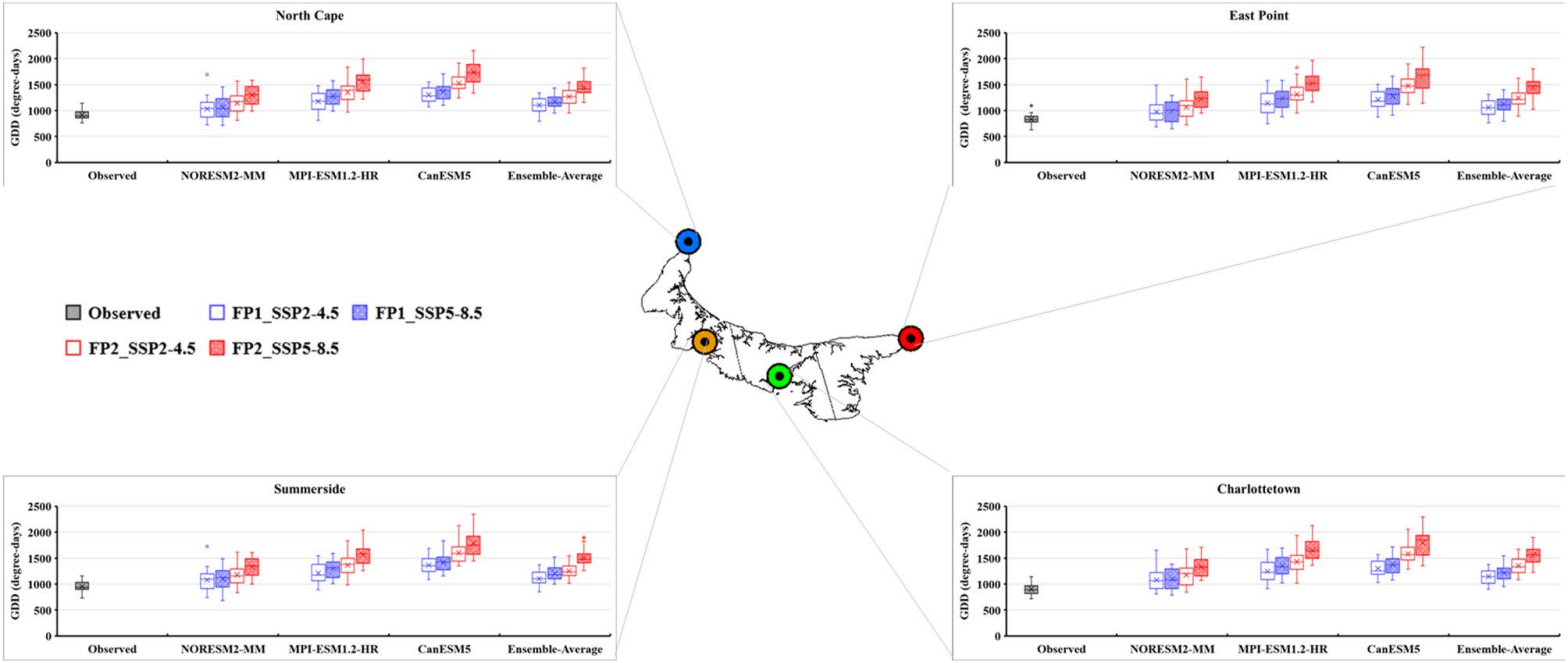
Projected variations in the average annual Growing Degree Days (degree-days) for baseline (1989–2014) and two future periods; FP1 (2026–2050), FP2 (2051–2075) at four selected stations (East Point, Charlottetown, Summerside, and North Cape) under SSP2-4.5 and SPP5-8.5 scenarios.

**Figure 13:**
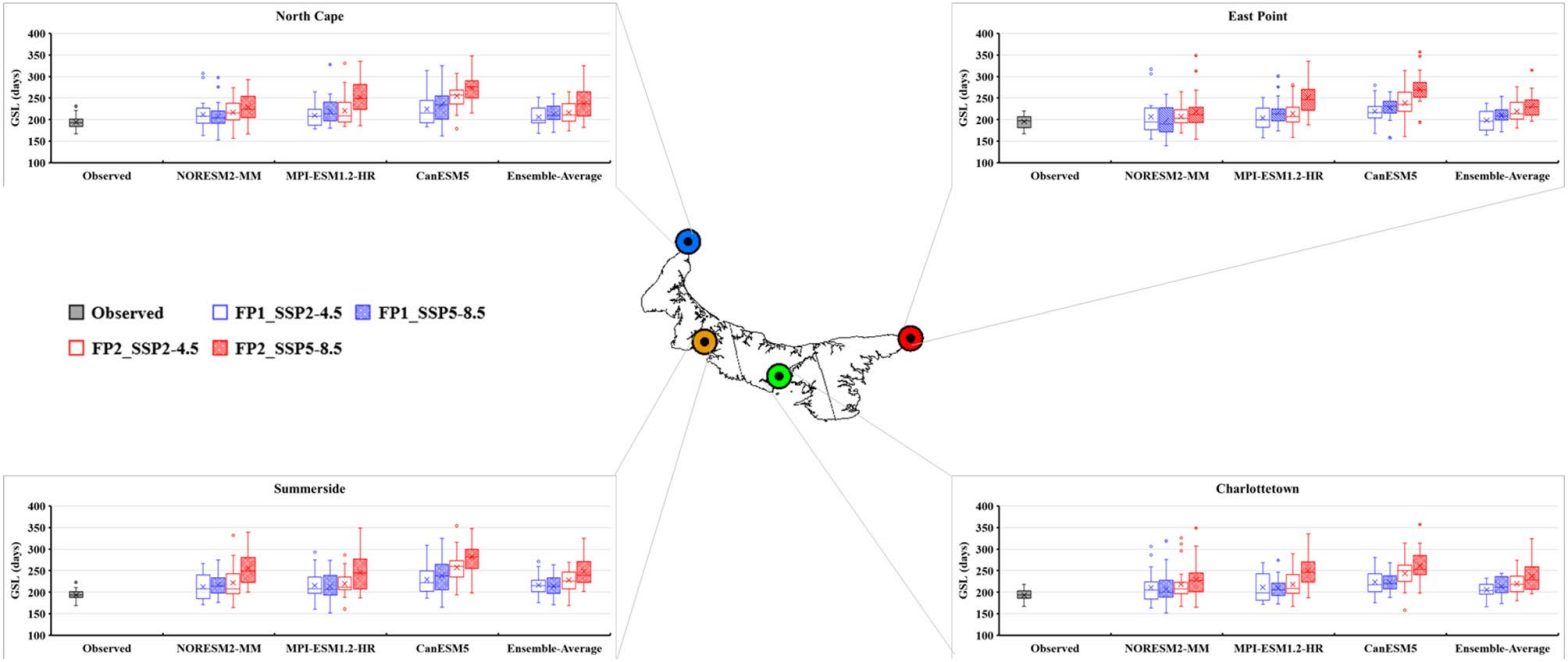
Projected variations in the average annual Growing Season Length (Days) for baseline (1989–2014) and two future periods; FP1 (2026–2050), FP2 (2051–2075) at four selected stations (East Point, Charlottetown, Summerside, and North Cape) under SSP2-4.5 and SPP5-8.5 scenarios. (*) asterisk sign indicates the outlier present in the data.

The bias-corrected results of three CMIP6 GCMs (NorESM2-MM, MPI-ESM-1.2-HR, and CanESM5) and their ensemble average were utilized to calculate the future temperature extreme indices under two different scenarios (SSP2-4.5 and SSP5-8.5). The future period was divided into two periods: FP1 (2026–2050) and FP2 (2051–2075). The projected changes in the six extreme temperature indices are presented as box-and-whisker plots (Figs. 8, 9, 10, 11, 12 and 13). Each box is defined in these plots by its upper and lower limits, which correspond to the 75th and 25th percentile values. The horizontal line within each box represents the median of the distributions. Additionally, the upper and lower whiskers of the plot indicate the 95th and 5th percentile values, respectively.

The DTR results revealed that NorESM2-MM exhibited the minimum difference between Tmax and Tmin, while the maximum for CanESM5 at all the stations due to their different ECS values at all the stations. East Point station depicted the lowest difference between Tmax and Tmin among the four stations. The average annual DTR projections, derived from the ensemble average of three models for future periods, varied from 6.76 to 7.30 °C at East Point, 8.93–9.48 °C at Charlottetown, 8.27–8.67 °C at Summerside, and 8.56–8.99 °C at North Cape, regardless of the SSPs (Fig. 8). An increasing trend was observed at all the stations and under all the GCMs. The highest rate of change per year in DTR (0.06–0.09 °C/year) was observed at East Point and North Cape stations, which are at the edge of the island, while in the middle two stations, DTR per year varied from 0.01 to 0.06 °C/year (Table 5). A wider DTR means warmer days and cooler nights, which result in higher evaporation rates and increased water demand. It can also induce stress in plants, which may impact crop yield. Pest attacks also increase with an increase in temperature. So, the DTR is essential for the farmers as it enables them to make decisions regarding crop selection, planting dates, irrigation management, pest control, and overall agricultural practices to maximize productivity, optimize resource utilization, and adapt to changing climate conditions.

The frost days (FD) are projected to decrease throughout the PEI, and the maximum decrease was observed during FP2 (Fig. 9). The lowest FD was observed for CanESM5, which has the highest ECS value among the selected three GCMs. These days are projected to decrease, according to the ensemble average of three models, from 141 (observed) to 127 (SSP2-4.5) and 96 days (SSP5-8.5) at East Point, 157 (observed) to 141 (FP1) and 112 days (FP2) at Charlottetown, 151 (observed) to 128 (SSP2-4.5) and 100 (SSP5-8.5) at Summerside, and 149 (observed) to 130 (SSP2-4.5) and 101 (SSP5-8.5) at North Cape during FP2 (Fig. 9). Furthermore, a decreasing trend was observed in FD for all the GCMs at every station. The annual reduction in FD, derived from the ensemble average of three GCMs, is projected to range from 0.00 to 1.00 days per year across the entire island (Table 4). The decrease in frost days has implications for agriculture on PEI as it extended the growing season. This increased growing season provides more opportunities for growing crops. On the other hand, the reduction in frost days may increase pest attacks and diseases. Hence, planting schedules, crop selection, and pest management strategies may be necessary to optimize agricultural production under the changing frost day patterns.

**Table 4:** Changes per year and trends of the Frost Days (FD), Summer Days (SU), and Ice Days (ID) for observed (1989–2014) and two future periods: FP1 (2026–2050) and FP2 (2051–2075), under two shared socioeconomic pathways SSP2-4.5 and SSP5-8.5.

Stations		FD (days/year)					SU (days/year)					ID (days/year)				
		OBS	FP1		FP2		OBS	FP1		FP2		OBS	FP1		FP2	
			SSP2-4.5	SSP5-8.5	SSP2-4.5	SSP5-8.5		SSP2-4.5	SSP5-8.5	SSP2-4.5	SSP5-8.5		SSP2-4.5	SSP5-8.5	SSP2-4.5	SSP5-8.5
East Point	Observed	↓0.89^c^					↑0.50^a^					↓0.75				
	NORESM2-MM		↓0.35	↓0.50	↓0.10	↓0.03		↑0.96^c^	↑1.10^b^	↑0.77	↑0.97^b^		↓0.68^c^	↓0.34	↓0.32^c^	↓↓0.54^c^
	MPI-ESM1.2-HR		↓1.08^c^	↓1.00^c^	↓0.00	0.60		↑1.68^a^	↑1.14^a^	↑0.87	↑1.30^a^		↓0.55	↓0.74^c^	↓0.33	↓0.17
	CanESM5		↓0.28	↓0.34	↓0.54	↓0.56		↑1.24^a^	↑1.26^a^	↑0.82^c^	↑1.45^a^		↓0.12	↓0.39^a^	↓0.20	↓0.57^c^
	Ensemble-Average		↓0.47	↓0.97^c^	0.00	0.08		↑1.5 9^a^	↑1.38^a^	↑1.13^c^	↑1.74^a^		↓0.68	↓0.50	↓0.23	0.00
Charlottetown	Observed	↓0.56					0.00					↓0.67				
	NORESM2-MM		↓0.17	↓0.58	↓0.03	↓0.26		↑0.85	↑0.21	↑0.11	↑0.49		↓0.63	↓0.32	0.00	↓0.56
	MPI-ESM1.2-HR		↓1.00^c^	↓1.14^c^	↓0.23	↓0.22		↑0.76	↑0.55	↑0.15	↑0.82^c^		↓0.65^c^	↓0.67	↓0.27	↓0.66
	CanESM5		↓0.70^c^	↓0.72^c^	↓0.73^c^	↓0.78		↑1.19^a^	↑0.75	↑0.54^a^	↑1.42^a^		↓0.05	↓0.50	↓0.25	↓0.57
	Ensemble-Average		↓0.83^c^	↓1.00^a^	↓0.50	↓0.67		↑0.62^c^	↑0.75	↑0.07	↑1.28^c^		↓0.50^a^	↓0.46	↓0.14	↓0.33
Summerside	Observed	↓0.31					↑0.27					↓0.09				
	NORESM2-MM		↓0.26	↓0.35	↓0.21	↓0.13		↑0.36	↑0.58	↑0.29	↑0.80		↓0.79	↓0.67	↓0.11	↓0.16
	MPI-ESM1.2-HR		↓0.80	↓0.57^c^	↓0.39	↓0.16		↑0.87^c^	↑0.73^c^	↑0.20	↑1.11^a^		↓0.73	↓0.75^a^	↓0.33	↓0.58^c^
	CanESM5		↓0.25	↓0.45	↓0.25	↓0.71		↑0.81^c^	↑0.71^c^	↑0.80	↑1.27^b^		↓0.23	↓0.36^c^	↓0.13	↓0.45
	Ensemble-Average		↓0.46	↓0.71^b^	↓0.76	↓0.50		↑0.76^c^	↑0.97^b^	↑0.46	↑1.25^a^		↓0.63^b^	↓0.51^a^	↓0.25	↓0.17
North Cape	Observed	↓1.12^a^					↑0.50^b^					↓0.28				
	NORESM2-MM		↓0.28	↓0.26	↓0.08	↓0.23		↑0.34	↑0.77	↑0.12	↑1.00		↓1.00^c^	↓0.69	↓0.12	↓0.27
	MPI-ESM1.2-HR		↓0.50	↓0.06	↓0.59	↓0.14		↑1.13^b^	↑0.71^b^	↑0.27	↑1.33^b^		↓0.53	↓1.00^b^	↓0.59	↓0.59
	CanESM5		↓0.26	↓0.08	↓0.38	↓0.52		↑1.00^c^	↑1.00	↑0.82	↑1.42^b^		↓0.22	↓0.48^a^	↓0.26	↓0.32
	Ensemble-Average		0.10	↓0.22	0.00	0.00		↑1.03^c^	↑1.19^a^	↑0.22	↑1.14^c^		↓0.65	↓0.53^a^	↓0.12	↓0.36

↑ Increasing trend, ↓ Decreasing trend, ^a^Significant at 0.001 level (99.9%); ^b^Significant at 0.01 level (99%); ^c^Significant at 0.05 level (95%).

The increase in summer days (SU) was higher during FP2 compared to FP1 across the entire island in all the GCMs. The increase in SU was observed in accordance with the ECS values of the GCMs. The results from the ensemble average of three GCMs showed that SU is projected to increase by 49 days at East Point, 46 days at Charlottetown, 44 at Summerside, and 48 days at North Cape compared to their observed average summer days during FP2 under SSP5-8.5 (Fig. 10). In the future, estimated summer days from the ensemble average data are projected to significantly increase from 0.07 to 1.74 days/year across the Island, irrespective of SSPs (Table 4). The increase in summer days may impact agriculture and other sectors of PEI. The farmers may need to adjust their planting schedules and irrigation practices to adapt to the extended summer period. Moreover, the rise in summer days may also reduce the energy consumption in winter, tourism patterns, and various other aspects of daily life on the island.

The projected increase in summer days on PEI is accompanied by a notable decrease in ice days (ID) at all the stations of PEI and a maximum decrease observed during FP2 (Fig. 11). The lowest number of IDs was observed for CanESM5, as it has a high ECS value and represents a higher temperature than the other two GCMs. Therefore, it reduces the occurrence of days where temperatures remain below freezing. The average annual number of ice days, based on the ensemble average of three models, is projected to decrease from 60 to 34 days at East Point, 63–35 days at Charlottetown, 48–36 days at Summerside, and 48–37 days at North Cape during FP2 under SSP5-8.5. The future projections suggest a decrease in ice days all over the island irrespective of the SSPs and GCMs, while the lowest ice days were observed with CanESM5 and the highest were observed with MPI-ESM1.2-HR. The ensemble average results of three GCMs for ice days show a decrement at all the stations ranging from 0.00 to 0.68 days/year, irrespective of SSPs (Table 4). The decrease in ice days can significantly affect various sectors, including transportation, infrastructure, and winter recreational activities.

Two agriculture-related indices, Growing degree days (GDD) and Growing season length (GSL), were also examined in this study. The results revealed that both indices exhibit an increase in future periods due to warming conditions. The GDD derived from the ensemble of three GCMs is projected to increase on an average from 835 (observed) to 1059 (SSP2-4.5) and 1126 degree-days (SSP5-8.5) during FP1, while during FP2, these days are projected to further increase to 1246 (SSP2-4.5) and 1452 degree-days (SSP5-8.5) at East Point. At Summerside, Average GDD is projected to increase from 940 (observed) to 1103 (SSP2-4.5) and 1208 degree-days (SSP5-8.5) during FP1 and 1255 (SSP2-4.5) and 1508 degree-days (SSP5-8.5) during FP2 at Summerside stations (Fig. 12). Yearly, All the GCMs projected an increment these days, with CanESM5 showing a maximum increment. The increment projected by the ensemble of three GCMs falls within the range of 5.41 to 15.58 degree-days across the island (Table 5). The increase in GDD has implications for agriculture on PEI. It provides an extended growing season, allowing for cultivating a wider range of crops and potentially enhancing crop yields. Farmers can utilize this information to optimize planting schedules, select appropriate crop varieties, and make decisions regarding agricultural practices.

**Table 5:** Changes per year and trends of the Daily Temperature Range (DTR), Growing Degree Days (GDD), and Growing Season Length (GSL) for observed (1989–2014) and two future periods: FP1 (2026–2050) and FP2 (2051–2075), under two shared socioeconomic pathways SSP2-4.5 and SSP5-8.5.

Stations		DTR (°C/year)					GDD (Degree-days/year)					GSL (days/year)				
		OBS	FP1		FP2		OBS	FP1		FP2		OBS	FP1		FP	
			SSP2-4.5	SSP5-8.5	SSP2-4.5	SSP2-4.5		SSP2-4.5	SSP5-8.5	SSP2-4.5	SSP5-8.5		SSP2-4.5	SSP5-8.5	SSP2-4.5	SSP5-8.5
East Point	Observed	↑0.08^a^					↑2.90					↑0.88^c^				
	NORESM2-MM		↑0.07^c^	↑0.07^c^	↑0.08^b^	↑0.07^b^		↑2.23	↑10.36	↑4.34	↑9.49^c^		↑1.38	↑2.39^c^	↑1.40^c^	↑0.37
	MPI-ESM1.2-HR		↑0.08^c^	↑0.09^b^	↑0.06^a^	↑0.09^c^		↑17.66^a^	↑11.25^c^	↑7.94	↑14.63^c^		↑1.23	↑1.73^b^	↑0.58	↑0.63
	CanESM5		↑0.07^b^	↑0.09^c^	↑0.08^b^	↑0.09^a^		↑13.18^c^	↑13.69^c^	↑10.32	↑16.75^b^		↑0.63	↑0.92	↑1.39^c^	↑1.67^a^
	Ensemble-Average		↑0.08^b^	↑0.09^b^	↑0.08^a^	↑0.09^b^		↑12.93^b^	↑12.13^a^	↑11.02^c^	↑15.58^a^		↑1.88^b^	↑0.98	↑2.04^a^	↑1.59^c^
Charlottetown	Observed	↑0.03^b^					↑5.78					↑0.72^c^				
	NORESM2-MM		↑0.01	↑0.01	↑0.02	↑0.02		↑0.64	↑6.69	↑3.30	↑4.78		↑0.66	↑1.25	↑1.38^a^	0.00
	MPI-ESM1.2-HR		↑0.02	↑0.02	↑0.02	↑0.02		↑16.10^c^	↑11.26	↑6.74	↑11.65^c^		↑0.54	↑1.27	↑1.00	↑0.94
	CanESM5		↑0.01	↑0.03	↑0.02	↑0.05^b^		↑8.42^a^	↑10.92^c^	↑7.93	↑17.13^c^		↑0.30	↑0.90	↑0.63	↑0.67
	Ensemble-Average		↑0.01	↑0.02	↑0.03	↑0.04^a^		↑10.66^a^	↑10.17^b^	↑6.59	↑14.48^a^		↑0.33	↑1.32	↑1.14^c^	↑1.27^c^
Summerside	Observed	↑0.01					↑3.49					↑0.09				
	NORESM2-MM		↑0.03^c^	↑0.04^c^	↑0.02^c^	↑0.04^b^		↑2.36	↑7.39	↑2.00	↑8.51		↑1.32	↑0.09	↑2.32^c^	↑0.03
	MPI-ESM1.2-HR		↑0.04	↑0.03	↑0.05^c^	↑0.03^c^		v11.74	↑7.61	↑5.38	↑11.43^c^		↑1.00	↑1.27	↑0.07	↑0.61
	CanESM5		↑0.05^a^	↑0.05^a^	↑0.06^a^	↑0.06^a^		↑6.79	↑7.51^a^	↑5.42	↑13.07^c^		↑0.49	↑1.17^c^	↑0.44	↑1.35
	Ensemble-Average		↑0.04^b^	↑0.04^b^	↑0.04^a^	↑0.05^a^		↑6.33	↑7.78^c^	↑4.29	↑12.05^b^		↑0.18	↑1.72^a^	↑1.38	↑2.31^b^
North Cape	Observed	↑0.06^a^					↑2.73					↑0.50^c^				
	NORESM2-MM		↑0.07	↑0.08	↑0.05	↑0.05		↑0.88	↑6.96	↑5.41	↑9.24		↑0.20	↑0.87	↑0.92	↑0.74
	MPI-ESM1.2-HR		↑0.08	↑0.09	↑0.05	↑0.08		↑13.42^c^	↑7.02	↑4.76	↑12.59^c^		↑0.73	↑0.29	↑0.76	↑0.54
	CanESM5		↑0.06	↑0.07	↑0.05	↑0.07		↑6.16	↑8.06	↑4.94	↑12.70		↑0.17	↑0.86	↑0.37	↑0.33
	Ensemble-Average		↑0.07	↑0.08	↑0.07	↑0.07		↑9.64^b^	↑9.40	↑5.41	↑11.71^b^		↑0.71	↑1.37^a^	↑0.42	↑2.20^b^

↑ Increasing trend, ↓ Decreasing trend, ^a^Significant at 0.001 level (99.9%); ^b^Significant at 0.01 level (99%); ^c^Significant at 0.05 level (95%).

The GSL also increased at all the stations of PEI and the results are in accordance with the ECS values of GCMs. The maximum growing season is projected in FP2 under SSP5-8.5 pathways of CanESM5 (Fig. 13). The eastern stations (East Point and Charlottetown) depicted maximum length for growing season in the future as compared to the western stations (North Cape and Summerside). The GSL from the ensemble average of three GCMs is projected to increase from 197 (observed) to 230 days (SSP5-8.5) at East Point, 194 (observed) to 229 days (SSP5-8.5) at Charlottetown, 194 (observed) to 239 days (SSP5-8.5) at Summerside, 193 (observed) to 237 days (SSP5-8.5) at North Cape during FP2. Furthermore, the change in GSL estimated by the ensemble average of three GCMs was observed to range from 0.18 to 2.31 days/year at all the stations, regardless of the emission scenarios (Table 5). The extension of the growing season length has important implications for agriculture on the island. It provides more time for crop growth. It also allows the cultivation of a wider range of crops and can lead to higher crop yields and diversification of agricultural practices. Farmers can utilize this information to optimize their planting and harvesting schedules, select appropriate crop varieties, and implement strategies to maximize the benefits of an extended growing season.

The results obtained in this study are consistent with the findings reported by Lu et al.^52^. In their study, they investigated temperature extreme indices for Toronto, Canada, and similarly observed increases in Tmax, Tmin, DTR, SU, GDD, and GSL. Additionally, they noted decreases in ID and FD, aligning with the trends observed in this current study for PEI.

### Monthly changes in climatic parameters

**Figure 14:**
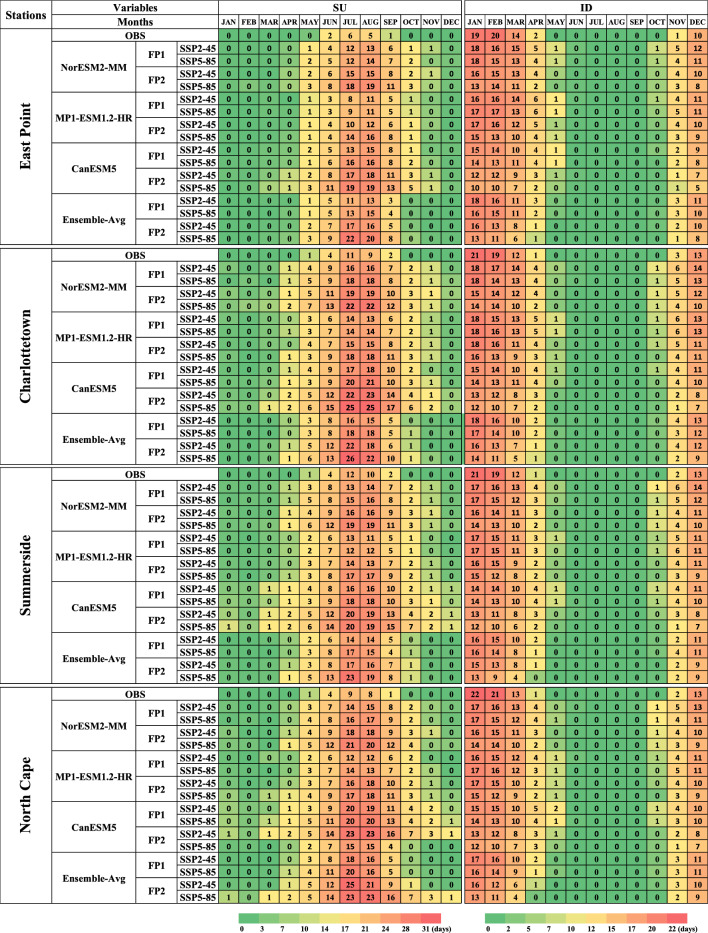
Projected changes in mean monthly Summer Days (SU) and Ice Days (ID) at East Point, Charlottetown, Summerside, and North Cape stations of Prince Edward Island during observed (1989–2014) and two future periods (FP1 (2026–2050) and FP2 (2051–2075)).

The projected changes to mean monthly SU and ID for observed and two future periods (FP1 and FP2) at all the stations under SSP2-4.5 and SSP5-8.5 are given in Fig. 14. The projected mean monthly Summer Days (SU) indicate that the summer months spanned from May to October during the observed period. However, in future projections, the summer period extends from May to November, extending the month count from six to seven. Additionally, at Charlottetown, Summerside, and North Cape stations, April and November are also included as a summer/warm month during FP2 under SSP5-8.5. The highest number of Summer Days (SU) was observed for CanESM5 during FP2 under the SSP5-8.5 scenario. The Ice Days (ID) indicated a decrease in the number of days in future periods compared to the observed period. In the observed period, Ice Days were observed from November to April, and similar changes were observed in the future periods. However, most of the ice days fall in December to March. Notably, the fewest Ice Days were observed for CanESM5 and during FP2 under the SSP5-8.5 scenario.

**Figure 15:**
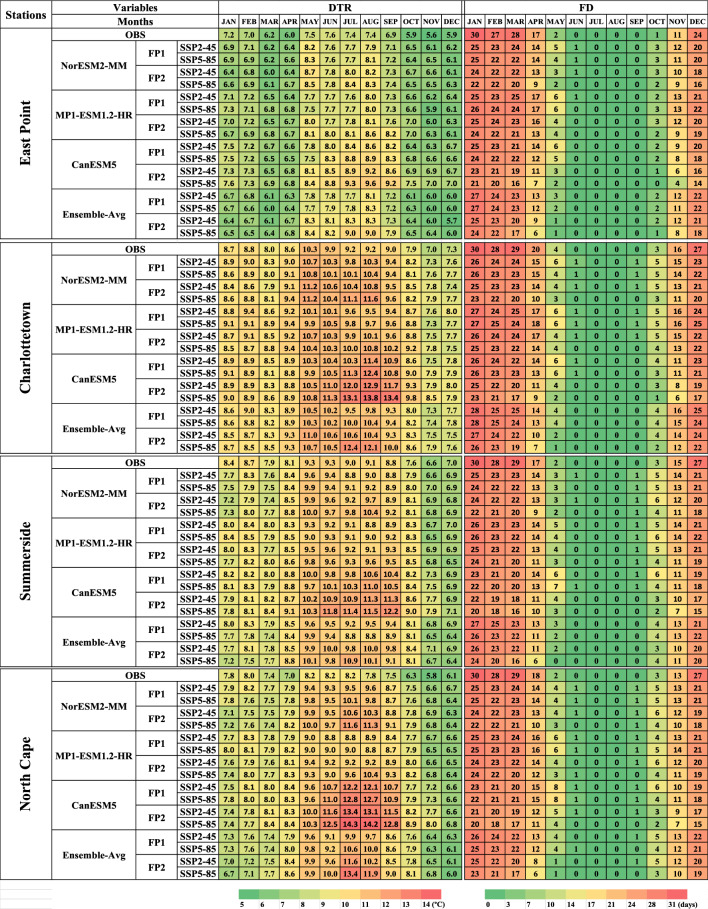
Projected changes in mean monthly Daily Temperature Range (DTR) and Frost Days (FD) at East Point, Charlottetown, Summerside, and North Cape stations of Prince Edward Island during observed (1989–2014) and two future periods (FP1 (2026–2050) and FP2 (2051–2075)).

The mean monthly results of DTR demonstrated less difference between Tmax and Tmin in the future periods at East Point station. Conversely, higher mean monthly DTR values were observed at all the stations, with North Cape and Charlottetown stations recording the maximum DTR. This suggests greater temperature fluctuations throughout the day or longer at these locations. It indicates that the island’s eastern part is colder than the central and western parts of PEI. Collectively, the results for DTR highlight that July, August, and September are expected to be the warmest months at all stations in the future periods under both SSP2-4.5 and SSP5-8.5 scenarios. The results for FD reveal that during the observed period, the months of June, July, August, and September had no frost days. However, the projected results for FP1 and FP2 indicate that June and September will also have a few frost days. Additionally, very few frost days are projected for the months of May and October. Notably, the fewest frost days are anticipated for CanESM5 during FP2 (Fig. 15).

## Conclusions

In this study, Stepwise Cluster Analysis (SCA) was employed to project changes in Tmax and Tmin at four stations on PEI (East Point, Charlottetown, Summerside, and North Cape). Two reanalysis datasets, ECMWF ERA5 and NCEP-DOE Reanalysis 2, were compared and evaluated for model calibration (1989–2006) and validation (2007–2014) periods. The results indicated that ECMWF ERA5 performed slightly better downscaling Tmax and Tmin than NCEP-DOE Reanalysis 2. As a result, ECMWF ERA5 was selected to develop a downscaling model. The Quantile delta mapping method was utilized to remove the biases from the model-simulated results for the historical and future periods. Three CMIP6 (NorESM2-MM, MPI-ESM1.2-HR, and CanESM5) GCMs, along with their ensemble average, were used to project Tmax and Tmin under two shared socioeconomic pathways (SSP2-4.5 and SSP5-8.5) during future periods (FP1 and FP2). These three GCMs were selected based on their different (low, medium, and high) equilibrium climate sensitivity values. These ECS values also impact the future projection of Tmax and Tmin, as low, medium, and high increments were observed for both Tmax and Tmin for NorESM2-MM, MPI-ESM1.2-HR, and CanESM5, respectively. Furthermore, the three GCMs and their ensemble average were used to calculate six temperature indices (DTR, FD, SU, ID, GDD, and GSL) to reduce any biases present in individual GCMs. The projected mean monthly changes revealed a warmer future climate for PEI. The DTR was observed to increase; months with frost days (FD) and ice days (ID) were projected to decrease, and months with summer days (SU) were projected to increase. Agriculture-related indices, such as growing degree days (GDD) and growing season length (GSL), were also projected to increase under both scenarios. The highest changes were observed for CanESM5, due to its high ECS value, during FP2 under SSP5-8.5 for all the temperature-related indices. The projected warming conditions have potential benefits for PEI growers, including extended summer and growing seasons. However, it is important to consider the potential impacts of rising sea levels and increased evapotranspiration rates, which contribute to more atmospheric moisture and potentially result in increased precipitation. The increase in evapotranspiration rate may also impact crop water requirements. Monitoring and analyzing these temperature indices can provide valuable insights for farmers and researchers, helping them assess crop suitability in specific regions, evaluate risks, optimize planting and harvesting schedules, and implement effective irrigation and crop protection strategies.

## Data Availability

The datasets used and analyzed during this study are available from Junaid Maqsood and the corresponding author (Xiuquan Wang) upon reasonable request. The contact details can be found at the beginning of this article.
